# Sulfatase 2-Induced Cancer-Associated Fibroblasts Promote Hepatocellular Carcinoma Progression via Inhibition of Apoptosis and Induction of Epithelial-to-Mesenchymal Transition

**DOI:** 10.3389/fcell.2021.631931

**Published:** 2021-04-06

**Authors:** Cong Wang, Chuzhi Shang, Xiaohong Gai, Tao Song, Shaoshan Han, Qingguang Liu, Xin Zheng

**Affiliations:** Department of Hepatobiliary Surgery, The First Affiliated Hospital of Xi’an Jiaotong University, Xi’an, China

**Keywords:** Sulf2, CAFs, HCC, OIP5-AS1/miR-153-3p, SDF-1/CXCR4

## Abstract

**Background:**

Sulfatase 2 (SULF2) removes the 6-*O*-sulfate groups from heparan sulfate proteoglycans (HSPG) and consequently alters the binding sites for various signaling molecules. Here, we elucidated the role of SULF2 in the differentiation of hepatic stellate cells (HSCs) into carcinoma-associated fibroblasts (CAFs) in the hepatocellular carcinoma (HCC) microenvironment and the mechanism underlying CAF-mediated HCC growth.

**Methods:**

The clinical relevance of SULF2 and CAFs was examined using *in silico* and immunohistochemical (IHC) analyses. Functional studies were performed to evaluate the role of SULF2 in the differentiation of HSCs into CAFs and elucidate the mechanism underlying CAF-mediated HCC growth. Mechanistic studies were performed using the chromatin immunoprecipitation, luciferase reporter, and RNA immunoprecipitation assays. The *in vitro* findings were verified using the nude HCC xenograft mouse model.

**Results:**

The Cancer Genome Atlas (TCGA) database and IHC analyses revealed that the expression of CAF markers, which was positively correlated with that of SULF2 in the HCC tissues, predicted unfavorable postsurgical outcomes. Co-culturing HSCs with HCC cells expressing SULF2 promoted CAF differentiation. Additionally, CAFs repressed HCC cell apoptosis by activating the SDF-1/CXCR4/PI3K/AKT signaling pathway. Meanwhile, SULF2-induced CAFs promoted epithelial-to-mesenchymal transition (EMT) of HCC cells by modulating the SDF-1/CXCR4/OIP5-AS1/miR-153-3p/SNAI1 axis. Studies using HCC xenograft mouse models demonstrated that OIP5-AS1 induced EMT by upregulating SNAI1 and promoted HCC growth *in vivo*.

**Conclusion:**

These data indicated that SULF2 secreted by the HCC cells induced the differentiation of HSCs into CAFs through the TGFβ1/SMAD3 signaling pathway. SULF2-induced CAFs attenuated HCC apoptosis by activating the SDF-1/CXCR4/PI3K/AKT signaling pathway and induced EMT through the SDF-1/CXCR4/OIP5-AS1/miR-153-3p/SNAI1 axis. This study revealed a novel mechanism involved in the crosstalk between HCC cells and CAFs in the tumor microenvironment, which can aid in the development of novel and efficient therapeutic strategies for primary liver cancer.

## Introduction

Hepatocellular carcinoma (HCC), which is associated with increased morbidity, is the second most common cause of cancer-related mortality worldwide (Ferlay et al., 2015; Llovet et al., 2021). The recent advances in novel immunotherapy (such as nivolumab; Yau et al., 2019; Pinter et al., 2021) and targeted therapy (such as sorafenib, Azzariti et al., 2016; Myojin et al., 2020; Levantine Hiraoka et al., 2019; or regorafenib, Teufel et al., 2019) have not markedly improved the overall survival of patients with HCC. Current therapeutic strategies for cancer are focused on the tumor cells and not on the cancer microenvironment. Various studies have suggested that the interaction between tumor cells and other components of the cancer microenvironment is critical for HCC progression. The HCC microenvironment comprises hepatic stellate cells (HSCs) (Teufel et al., 2019; Myojin et al., 2020), carcinoma-associated fibroblasts (CAFs) (Affo et al., 2017; Yang et al., 2020), immune cells (Wilson et al., 2015; Cadoux et al., 2021), growth factors (Kim et al., 2019), proteolytic enzymes, cytokine, and extracellular matrix (ECM) (Filliol and Schwabe, 2019). CAFs in the cancer stroma tissue secrete growth factors and cytokines, recruit immunosuppressive cells, modify ECM, and contribute to HCC progression (Giannelli et al., 2014; Zhou et al., 2018; Baglieri et al., 2019). Approximately 90% of HCC cases develop from advanced liver fibrosis or cirrhosis. Hence, CAFs have a critical role in the fibrotic microenvironment to promote hepatocarcinogenesis (Baglieri et al., 2019). Several studies have demonstrated that the CAFs are derived from diverse sources, including epithelial-to-mesenchymal transition (EMT) of tumor cells (Fukino et al., 2004) and bone marrow mesenchymal cells (Raz et al., 2018). In the HCC microenvironment, HSCs frequently differentiate into CAFs (Song et al., 2015; Zheng et al., 2016; Fang et al., 2018; Luo et al., 2018). However, the mechanism underlying the differentiation of HSCs into CAFs has not been elucidated.

Extracellular sulfatase 2 (SULF2), which belongs to the sulfatase family, selectively removes 6-*O*-sulfate groups from glucosamine residues within the heparan sulfate (HS) chains and modulates multiple molecular processes in the cancer microenvironment (Hammond et al., 2014; Zizza et al., 2019). Previous studies have reported that SULF2 regulates cancer progression by modulating the cell signaling pathways through two mechanisms. SULF2 can modify the interactions between signaling ligands and their cognate receptors by regulating the HS component of the ECM (Kirn-Safran et al., 2008; Yuzhalin et al., 2018). Alternatively, SULF2 can regulate the HS-dependent signaling pathway through the modulation of heparan sulfate proteoglycan (HSPG) expression (Rosen and Lemjabbar-Alaoui, 2010; Chen et al., 2020). SULF2, which is reported to be an unfavorable prognostic factor for patients with HCC, promotes HCC progression by regulating various cell signaling pathways, including the TGFβ1/SMAD (Zheng et al., 2013; Chen et al., 2017), PI3K/ERK (Lai et al., 2010b; Bao et al., 2013), Hedgehog/GLI1 (Zheng et al., 2013), GPC3/Wnt (Lai et al., 2010a), and GPC3/FGF2 signaling pathways (Lai et al., 2008). However, the correlation between SULF2 and CAFs in the HCC microenvironment has not been elucidated.

Previously, we had reported that the SULF2 inhibitor 2,4-disulfonylphenyl-*tert*-butylnitrone (OKN-007) suppressed HCC growth by downregulating the TGFβ1/SMAD1 signaling pathway. TGFβ1 is reported to promote the differentiation of fibroblasts into CAFs (Calon et al., 2014; Yao et al., 2018; Tang et al., 2019). The SDF-1/CXCR4 signaling pathway promotes the development of HCC by facilitating tumor growth, angiogenesis, and HCC metastases (Song et al., 2015; Wang et al., 2016). The upregulated level of CXCR4 in the tumor tissues was significantly correlated with poor prognosis in patients with HCC (Hu et al., 2015; Neve Polimeno et al., 2015). Some studies have demonstrated that CAFs secrete SDF-1 in the tumor microenvironment of various cancers, including pancreatic carcinoma (Wei et al., 2018; Broekgaarden et al., 2019), colorectal cancer (Peng et al., 2018; Kasashima et al., 2021), and lung cancer (Zhu et al., 2017; Shen et al., 2020). This study aimed to determine the role of SULF2, which was upregulated in the HCC microenvironment, in promoting the differentiation of HSCs into CAFs through the TGFβ1/SMAD2 signaling pathway. The findings of this indicated that CAFs suppressed HCC cell apoptosis by activating the SDF-1/CXCR4/PI3K/AKT pathway.

## Subjects and Methods

### Study Subjects and Tissue Specimens

In total, 102 HCC tissues and pair-matched adjacent liver tissues were obtained from patients with HCC undergoing curative liver resection at the Department of Hepatobiliary Surgery of the First Hospital of Xian Jiaotong University between 2015 and 2016. The clinicopathological data of the study cohort are summarized in Supplementary Table 1. None of the patients in the study cohort underwent transcatheter arterial chemoembolization, radiofrequency ablation, and systemic treatment before surgery. All harvested tissue specimens were frozen in liquid nitrogen and stored at −80°C for RNA isolation. The histopathological features of HCCs were determined by two experienced pathologists. The study protocols involving human specimens were approved by the Committee for Ethical Review of Research involving Human Subjects of the First Hospital of Xian Jiaotong University (XJTU1AF2015LSL-024). Informed consent was obtained from all patients before surgery to collect the tissue.

### Cell Lines and Cell Culture

The human HCC cell lines (Hep3B, PLC/PRF/5, and SNU398), non-HCC cell line (LO2), and the HSC cell line (LX2 cells) were purchased from American Type Culture Collection (Manassas, VA, United States). The cells used in all experiments were at passages 4–6. The cells were passaged for less than 6 months in our laboratory. Huh7 cells were obtained from the Japan Health Science Research Resources Bank (Osaka, Japan). All cells were cultured in Dulbecco’s modified Eagle medium (Gibco, United States) supplemented with 10% fetal bovine serum in a humidified incubator at 37°C and 5% CO_2_. The major reagents and kits used in the study are summarized in Supplementary Table 2.

### Quantitative Real-Time Polymerase Chain Reaction

Total RNAs from the HCC tissues and cell lines were extracted using TRIzol reagent (Invitrogen, Carlsbad, CA, United States). The isolated RNA (1 μg) was reverse transcribed into complementary DNA using the high-capacity cDNA reverse transcription kit (Applied Biosystems, Carlsbad, CA). The qRT-PCR analysis was performed using the Premix ExTaq II kit (Takara). We diluted the cDNA to one-tenth of the original concentration within each sample, and then we took 2 μl of the diluted cDNA and added it to the 25 μl reaction system for PCR quantification (the amount of cDNA added was below 50 ng). The sequences of the primers used for qRT-PCR analysis and the quantity of RNA are summarized in Supplementary Table 3. The expression levels of the target genes were normalized to those of *ACTB* (beta-actin), and the expression levels of genes were calculated using the 2^–ΔΔCt^ method.

Differences were calculated using the Ct and comparative Ct methods for relative quantification. The relative level of gene expression was normalized with a housekeeping gene *ACTB* (*beta-actin*) and was used as a reference to calculate the relative level of gene expression using the formula 2^–ΔΔCt^.

### Establishment of Sulfatase 2 Overexpressing Hepatocellular Carcinoma Clones

The full-length *SULF2* cDNA was cloned into the pcDNA3.1 plasmid to overexpress SULF2. The sequences of short hairpin RNA (shRNA) against SULF2 mRNA (sh-SULF2) were as follows: shRNA-1, AAGTACGTCCACAACCACA; shRNA-2, AATGTGACTGTCACAAAAT. The shRNAs were cloned into the vector pSS-H1p. The cells were transfected with different plasmids as described previously (Zheng et al., 2013). Briefly, the vector pSS-H1p containing scrambled target sequence was used as controls. Cells were grown to 80–90% confluence and transfected with SULF2-expressing plasmid using FuGENE^®^ 6 Transfection Reagent from Roche (Indianapolis, IN). Geneticin (G418) from Invitrogen (Carlsbad, CA) at a dose of 300 μg/ml was used to select stably SULF2-expressing clones. Similarly, cells were transfected with both SULF2 shRNA-a and shRNA-b synchronously using FuGENE^®^ 6. The stable transfectant clones were obtained after 2-week selection with 600 μg/ml of G418 (cells used for this experiment were at passages 4–6).

### Cell Migration and Invasion Assays

Cell migration was examined using the wound-healing assay as described previously (Liu et al., 2017). The invasion ability of HCC cells was examined using Transwell chambers coated with Matrigel (Liu et al., 2017). The HCC cells (5 × 10^4^ cells) were seeded in the upper chamber containing serum-free medium. The bottom chamber comprised medium supplemented with 10% fetal bovine serum. The cells were incubated at 37°C for 24 h (migration) or 48 h (invasion). Next, the cells on the membrane were immobilized and stained with crystal violet. The results were analyzed by counting the stained cells using optical microscopy in five randomly selected fields (cells used for this experiment were at passages 4–6).

### Immunofluorescence and Confocal Microscopy

The LX2 cells (3 × 10^4^) were cultured on Lab-Tek II Chamber Slides for 24 h and fixed with 2.5% formaldehyde in PIPES buffer for 20 min. The cells were rinsed with phosphate-buffered saline (PBS) at room temperature and incubated with blocking buffer (5% normal goat serum and 5% glycerol in PBS) for 1 h at 37°C. Further, the cells were incubated with anti-TGFβ1 (Catalog No. 3711, Cell Signaling Technology) antibodies overnight at 4°C. The cells were then washed with PBS and incubated with the fluorescein isothiocyanate (FITC)-labeled secondary antibodies for 2 h at room temperature. Finally, the cells were rinsed with PBS, stained with 4′,6-diamidino-2-phenylindole (DAPI), and subjected to confocal microscopy analysis (cells used for this experiment were at passages 4–6).

### Western Blotting and Immunohistochemical Analyses

Total proteins were extracted from the HCC and LX2 cells, quantified, and subjected to western blotting analysis using the anti-TGFB1 (#3711, Cell Signaling Technology), anti-p-SMAD3 (#9520, Cell Signaling Technology), anti-SDF-1 (#3530, Cell Signaling Technology), anti-CXCR4 (#997680, Cell Signaling Technology), anti-p-PI3K (#4228, Cell Signaling Technology), anti-p-AKT (#4060, Cell Signaling Technology), anti-p-BAD (#5284, Cell Signaling Technology), anti-p-CASP9 (#AP0108, Bioworld), anti-p-FKHRL 1 (#9466, Cell Signaling Technology), anti-SNAI1 (#3879, Cell Signaling Technology), anti-CDH1 (#3195, Cell Signaling Technology), anti-CDH2 (#13116, Cell Signaling Technology), anti-VIM (#5741, Cell Signaling Technology), anti-SULF2 (#PA5-43331, Thermo Fisher Scientific), and anti-ACTB antibodies (#3700, Cell Signaling Technology), following the previously reported standard protocols (Wang C. et al., 2019). The HCC specimens and HCC xenografts were subjected to immunohistochemical (IHC) analysis (Yang et al., 2017) using the anti-SULF2 (#PA5-43331, Thermo Fisher Scientific), anti-FAP (#66562, Cell Signaling Technology), anti-ACTA2 (#19245, Cell Signaling Technology), anti-POSTN (#91771, Cell Signaling Technology), anti-SNAI1 (#3879, Cell Signaling Technology), anti-CDH1 (#3195, Cell Signaling Technology), and anti-VIM antibodies (#5741, Cell Signaling Technology). The details of the antibodies are summarized in Supplementary Table 2.

### Luciferase Reporter Assay

The recombinant OIP5-AS1 luciferase reporter plasmids were generated by cloning miR-153-3p binding sites (OIP5-AS1-WT) or mutant type (OIP5-AS1-MUT) into the pRL Renilla luciferase control reporter vector. Briefly, the HCC cells (60–90% confluency) were transfected with recombinant luciferase reporter plasmids in 48-well plates using Lipofectamine 2000 (Invitrogen). At 48 h post-transfection, the luciferase activity in the HCC cell lysate was examined using the Dual-Glo^®^ luciferase assay system (Promega) (cells used for this experiment were at passages 4–6).

### Microarray Analysis

The mRNA expression profiles of the SULF2-transfected and vector-transfected LX2 cells were examined using HiSeq X ten PE150NovaSeq 6000 from GeneChem (cells used for this experiment were at passages 4–6).

### Chromatin Immunoprecipitation Assay

The control cells and TGFβ1-treated LX2 cells were fixed with 1% formaldehyde for 10 min at room temperature. The cells were then subjected to chromatin immunoprecipitation (ChIP) assay using the Pierce^TM^ agarose ChIP kit, following the manufacturer’s instructions. The anti-p-SMAD3 antibody was purchased from Cell Signaling Technology (#9520). The following primers were used for the analysis: *ACTA2* promoter, 5′-TTTGACAGGCTGCTGGGTAG-3′ (forward) and 5′-TCCACTGCATTCCACTGGTC-3′ (reverse); *POSTN* promoter, 5′-TCCTTCACCTCCAGTCAAACC-3′ (forward) and 5′-ACACTGCTACTAATGCCACACT-3′ (reverse) (cells used for this experiment were at passages 4–6).

### RNA Immunoprecipitation

RNA immunoprecipitation (RIP) assay was performed to determine whether OIP5-AS1 functioned as a competing endogenous RNA (ceRNA) to sequester miR-153-3p using RIP^TM^ RNA-binding protein immunoprecipitation kit from Merck Millipore, following the manufacturer’s instructions. Briefly, cells were collected and lysed in complete RIP lysis buffer. Then, the cell extract was incubated with RIP buffer containing magnetic beads conjugated to a human anti-Ago2 antibody (Millipore, United States). Samples were incubated with proteinase K with shaking to digest proteins, and the immunoprecipitated RNA was isolated. IgG was used as a negative control. The results of RIP were analyzed using qRT-PCR (cells used for this experiment were at passages 4–6).

### Cell Apoptosis Analysis

For flow cytometric analysis, the Hep 3B cells were cultured in 6-well plates at an initial density of 2.5 × 105 cells/well and then incubated with or without CAFs and AMD3100. After the incubation, the cells were subjected to staining with propidium iodide (PI) (10 μg/ml; BD Biosciences) and FITC−conjugated annexin V (annexin V–FITC; BD Biosciences) for 15 min at room temperature in the darkness. Cell apoptosis was analyzed using a flow cytometer (FACSCalibur; BD Biosciences) (cells used for this experiment were at passages 4–6).

For caspase−3/7 activity (Abcam, United States), the Hep 3B cells (50,000 cells) were stained for the cleaved or active form of caspase-3/7 marker fluorescence dye. Cells treated with 10 μl of PBS were used as untreated control. After incubation with or without CAFs, cells were washed twice using 100 μl of PBS and stained with 5 μM of caspase-3/7 red fluorescence apoptosis marker in 100 μl of the respective medium. The cells were incubated for 30–45 min at 37°C. The fluorescence data were detected for 20,000 events/sample and acquired using a flow cytometer (FACSCalibur; BD Biosciences). The bandpass filters used in the flow cytometry were 530/30 and 585/40. The laser excitation wavelength was 488 nm (cells used for this experiment were at passages 4–6).

### TdT-Mediated dUTP Nick-End Labeling Assay

Tissues were fixed in 4% paraformaldehyde and embedded in paraffin. Five-micron sections were obtained after deparaffinization and rehydration. To assess the levels of apoptosis of cells, paraffin sections were stained using TdT-mediated dUTP nick-end labeling (TUNEL) apoptosis assay kit (Roche, Germany) according to the manufacturer’s protocol. The nuclei of apoptosis cells were then stained in brownish-yellow, while the nuclei of normal cells were stained in blue. The stained sections were then observed under the Nikon LV150N optical microscope (Nikon, Tokyo, Japan).

### Hepatocellular Carcinoma Xenograft Experiments

All *in vivo* protocols were approved by the Institutional Animal Care and Use Committee of the First Affiliated Hospital of Xi’an Jiaotong University. The animal studies were approved by the research ethics committee of the First Affiliated Hospital of Xi’an Jiaotong University (reference number. XJTU1AF2015LSK-225). Male nude mice aged 4–6 weeks were randomly divided into the following two groups: SNU398 OIP5-AS1 siRNA group, subcutaneously injected with OIP5-AS1 siRNA-transfected SNU398 cells (5 × 10^6^); and SNU398 Scr siRNA (5 × 10^6^) group, subcutaneously injected with scramble (Scr) siRNA-transfected SNU398 cells (5 × 10^6^) and washed with PBS reconstituted with Matrigel (1:10; BD Biosciences) in MEM to a final volume of 0.1 ml/mouse. The volume of the HCC xenografts was measured every week using the following formula: volume = A × B^2^ × 0.52 (A, length; B, width). On day 28 post-HCC cell injection, all xenografts were harvested and subjected to qRT-PCR and IHC analyses.

### Statistical Analysis

All statistical analyses were performed using GraphPad Prism 8.0 software (GraphPad Inc.). The means of two groups were compared using Student’s *t*-test or Mann–Whitney *U*-test. At least three independent experiments were performed. The Kaplan–Meier survival curves were analyzed using the log-rank test. The differences were considered significant at *P* < 0.05.

## Results

### Carcinoma-Associated Fibroblast Markers Were Positively Associated With Sulfatase 2 in the Hepatocellular Carcinoma Tissues and Predicted Unfavorable Surgical Outcomes

**FIGURE 1:**
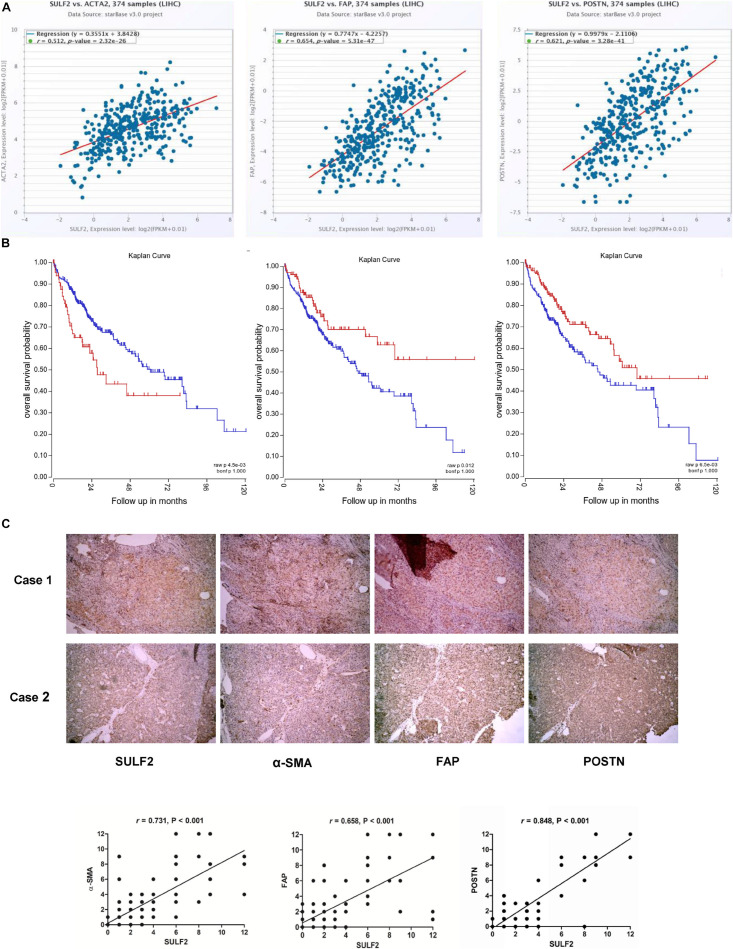
SULF2 expression in HCC tissues was related positively with CAF markers, which predicted poor prognosis after surgery. **(A)** TCGA database showed that SULF2 mRNA in HCC tissues was significantly associated positively with α-SMA (*r* = 0.512, *P* < 0.01), FAP (*r* = 0.654, *P* < 0.01), and POSTN (*r* = 0.512, *P* < 0.01). **(B)** The further analysis of TCGA database displayed that CAF markers, including α-SMA (*P* < 0.0045), FAP (*P* = 0.012), and POSTN (*P* = 0.006), were significantly associated with the worse prognosis of HCC patients. **(C)** IHC staining assay about 102 HCC samples collected from our hospital showed that SULF2 expression was positively associated with α-SMA (*r* = 0.731, *P* < 0.001), FAP (*r* = 0.658, *P* < 0.001), and POSTN (*r* = 0.848, *P* < 0.01), respectively. SULF2, sulfatase 2; HCC, hepatocellular carcinoma; CAF, carcinoma-associated fibroblast; TCGA, The Cancer Genome Atlas; IHC, immunohistochemical.

The correlation between SULF2 and CAFs in the HCC microenvironment was examined based on The Cancer Genome Atlas (TCGA) database and IHC analyses of HCC tissues harvested during liver resection. As shown in Figure 1A, TCGA database analysis revealed that the mRNA expression level of *SULF2* in the HCC tissues was significantly and positively correlated with that of *ACTA2* (actin alpha 2, smooth muscle; *r* = 0.512; *P* < 0.01), *FAP* (fibroblast activation protein; *r* = 0.654; *P* < 0.01), and *POSTN* (periostin; *r* = 0.512; *P* < 0.01), which are considered as CAF biomarkers (Figure 1A). Additionally, the mRNA expression level of *SULF2* was significantly upregulated in the HCC tissues (Supplementary Figure 1). This indicated that *SULF2* mRNA expression was positively correlated with CAFs in the HCC microenvironment and that *SULF2* upregulation may contribute to CAF differentiation. Furthermore, TCGA database analysis revealed that *ACTA2*, *FAP*, and *POSTN* were significantly correlated with poor prognosis in patients with HCC (Figure 1B). These data indicated that the increased number of CAFs is positively correlated with unfavorable prognosis in patients with HCC.

IHC analysis of 102 HCC samples was performed to examine the correlation between SULF2 and CAFs at the protein level. As shown in Figure 1C, the protein expression levels of SULF2 were significantly and positively correlated with those of ACTA2, FAP, and POSTN in the HCC tissues.

### Hepatic Stellate Cells Differentiated Into Carcinoma-Associated Fibroblasts Upon Co-culturing With Hepatocellular Carcinoma Cells Expressing Sulfatase 2

**FIGURE 2:**
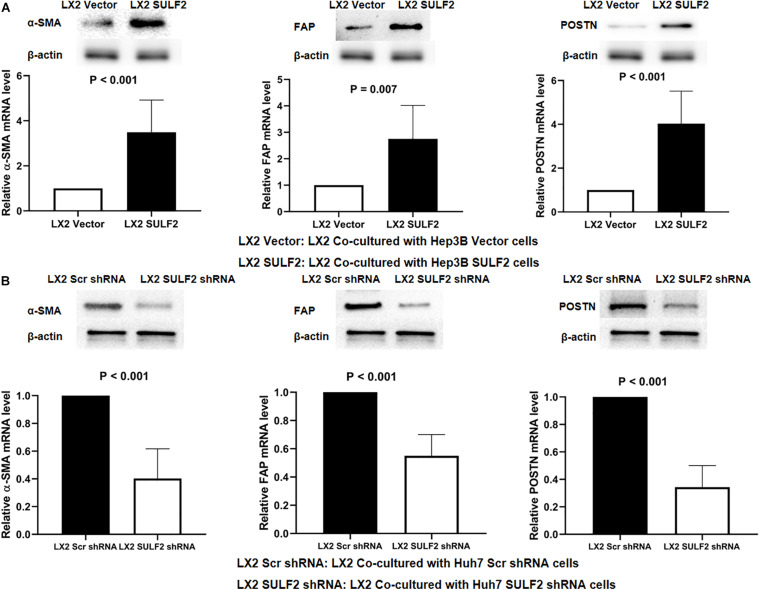
SULF2 secreted by HCC cells induced the transformation from HSCs to CAFs. **(A)** Both RT-PCR and western immunoblotting assays showed that there was more expression of α-SMA, FAP, and POSTN in LX2 cells co-cultured with Hep3B SULF2 cells (LX2 SULF2 cells) than those co-cultured with Hep3B Vector cells (the protein was extracted from total cells). **(B)** LX2 cells co-cultured with Huh7 Scr shRNA cells expressed more α-SMA, FAP, and POSTN than those with Huh7 SULF2 shRNA cells. Cell fractions: LX2 cells (the protein was extracted from total cells). SULF2, sulfatase 2; HCC, hepatocellular carcinoma; CAF, carcinoma-associated fibroblast.

Previous studies have demonstrated that tumor cells secreted increased amounts of SULF2 in the HCC microenvironment (Lai et al., 2010a; Zheng et al., 2013; Chen et al., 2017). To examine the effect of SULF2 secreted by the tumor cells on HSCs in the HCC microenvironment, a SULF2 overexpressing Hep3B cell line was established (Supplementary Figure 2A). The SULF2 overexpressing Hep3B cells were co-cultured with LX2 cells for 72 h. The results of the enzyme-linked immunosorbent assay (ELISA) revealed that the protein expression level of SULF2 in the culture medium of SULF2-transfected Hep3B cell/LX2 cell co-culture was significantly higher than that in the culture medium of vector-transfected Hep3B cell/LX2 cell co-culture (Supplementary Figure 2B). The qRT-PCR and western blotting analyses revealed that the expression levels of ACTA2, FAP, and POSTN in the LX2 cells co-cultured with SULF2-transfected Hep3B cells were markedly higher than those in the LX2 cells co-cultured with vector-transfected Hep3B cells (Figure 2A). To validate this finding, *SULF2* was silenced in the Huh7 cells using the sh-SULF2 constructs (Supplementary Figure 2C). The protein expression level of SULF2 in the sh-SULF2-transfected Huh7 cells was lower than that in the Scr shRNA-transfected Huh7 cells (Supplementary Figure 2D). Additionally, the expression levels of CAF markers in the LX2 cells co-cultured with Scr shRNA-transfected Huh7 cells were higher than those in the LX2 cells co-cultured with sh-SULF2-transfected Huh7 cells (Figure 2B). These findings demonstrated that SULF2 secreted by the HCC cells promote the differentiation of LX2 cells into CAFs.

**FIGURE 3:**
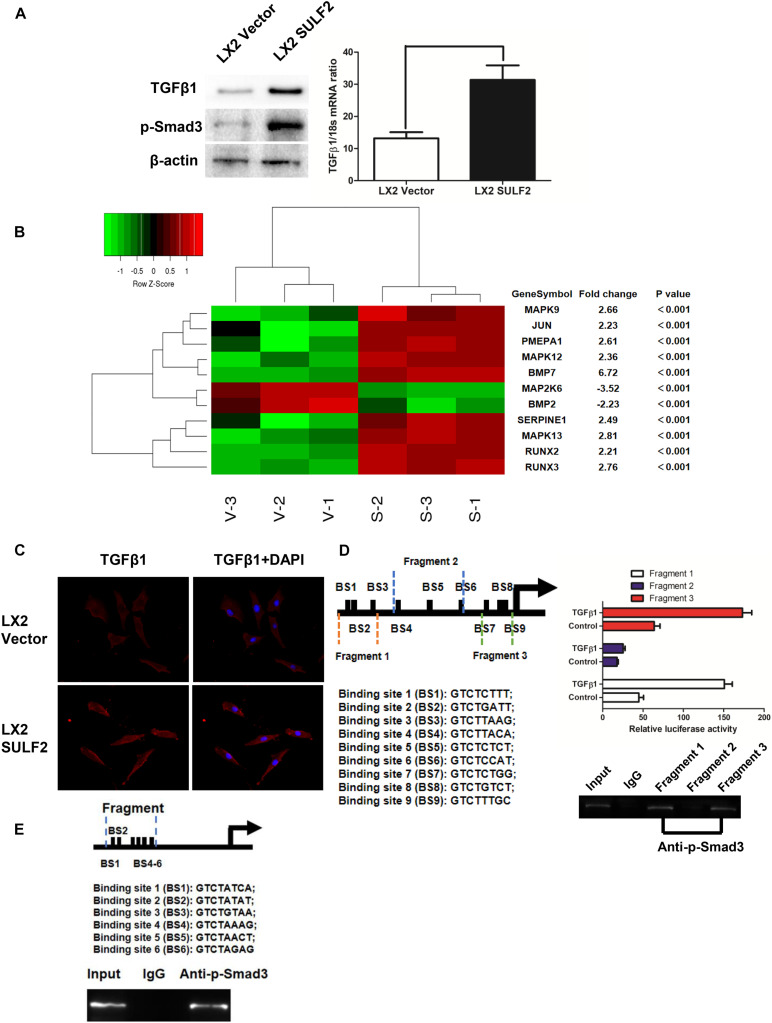
SULF2 activated HSCs to CAFs via mediating TGFβ1/SMAD3 signaling. **(A)** LX2 cells co-cultured with Hep3B SULF2 cells (LX2 SULF2 cells) were found to express more expression of both TGFβ1 and p-SMAD3 than those with Hep3B Vector cells (LX2 Vector cells) by western immunoblotting and RT-PCR. Cell fractions: LX2 cells (the protein was extracted from total cells). **(B)** Microarray profiling of mRNA expression showed that there was a significantly higher mRNA expression of TGFβ1 signaling downstream genes including BMP7, MAPK9, JUN, PMEPA1, MAPK12, SERPINE1, MAPK13, RUNX2, and RUNX3 in LX2 SULF2 cells than LX2 Vector cells. **(C)** Immunofluorescence staining displayed that more TGFβ1 protein was bound in the cell membrane of LX2 SULF2 cells than LX2 Vector cells. **(D)** Both luciferase reporter and ChIP assays confirmed that p-SMAD3 was bound with the α-SMA promoter directly in LX2 cells. **(E)** ChIP assay showed that p-SMAD3 protein was able to bind with the promoter of POSTN in LX2 cells treated with TGFβ1. SULF2, sulfatase 2; HCC, hepatocellular carcinoma; CAF, carcinoma-associated fibroblast; ChIP, chromatin immunoprecipitation.

To elucidate the mechanism underlying SULF2-mediated CAF differentiation, the correlation between the expression of SULF2 and TGFβ1/SMAD3 signaling was examined. The expression level of TGFβ1 and p-SMAD3 in the SULF2-transfected LX2 cells was significantly higher than that in the vector-transfected LX2 cells (Figure 3A). Microarray analysis was performed to examine the activation of the TGFβ1 signaling pathway in the SULF2-transfected and vector-transfected LX2 cells. As shown in Figure 3B, the mRNA expression levels of TGFβ1 signaling-related genes, including *BMP7*, *MAPK9*, *JUN*, *PMEPA1*, *MAPK12*, *SERPINE1*, *MAPK13*, *RUNX2*, and *RUNX3*, in the SULF2-transfected LX2 cells, were significantly higher than those in the vector-transfected LX2 cells. Immunofluorescence staining revealed that the membrane expression of TGFβ1 protein in the SULF2-transfected LX2 cells was higher than that in the vector-transfected LX2 cells (Figure 3C). This suggested that SULF2 could activate the TGFβ1/SMAD3 pathway by promoting the interaction between TGFβ1 and TGF receptors.

*In silico* analysis revealed nine potential SMAD-binding sites in the promoter region of *ACTA2*. As shown in Figure 3D, the following three DNA fragments containing different regions of the *ACTA2* promoter were constructed: fragment 1 contained the −1,958- to −1515-bp region; fragment 2 contained the −1,083- to −553-bp regions; and fragment 3 contained the −293- to −42-bp region. Fragments 1, 2, and 3 were cloned into the pGL3 reporter vector. The recombinant pGL3 vectors were transfected into the LX2 cells. The results of the dual-luciferase reporter assay revealed that TGFβ1 treatment increased the luciferase activity of both pGL3-fragment 1 and pGL3-fragment 3 constructs. Furthermore, the results of the ChIP assay revealed that SMAD3 protein binds to both fragment 1 and fragment 2 (Figure 3D). Bioinformatic analysis revealed six potential SMAD-binding sites in the promoter region of *POSTN* (Figure 3E). The results of the ChIP assay also verified that p-SMAD3 protein could bind to the promoter of *POSTN* in the TGFβ1-treated LX2 cells.

### Carcinoma-Associated Fibroblast Repressed Hepatocellular Carcinoma Cell Apoptosis by Activating the SDF-1/CXCR4/PI3K/AKT Signaling Pathway

**FIGURE 4:**
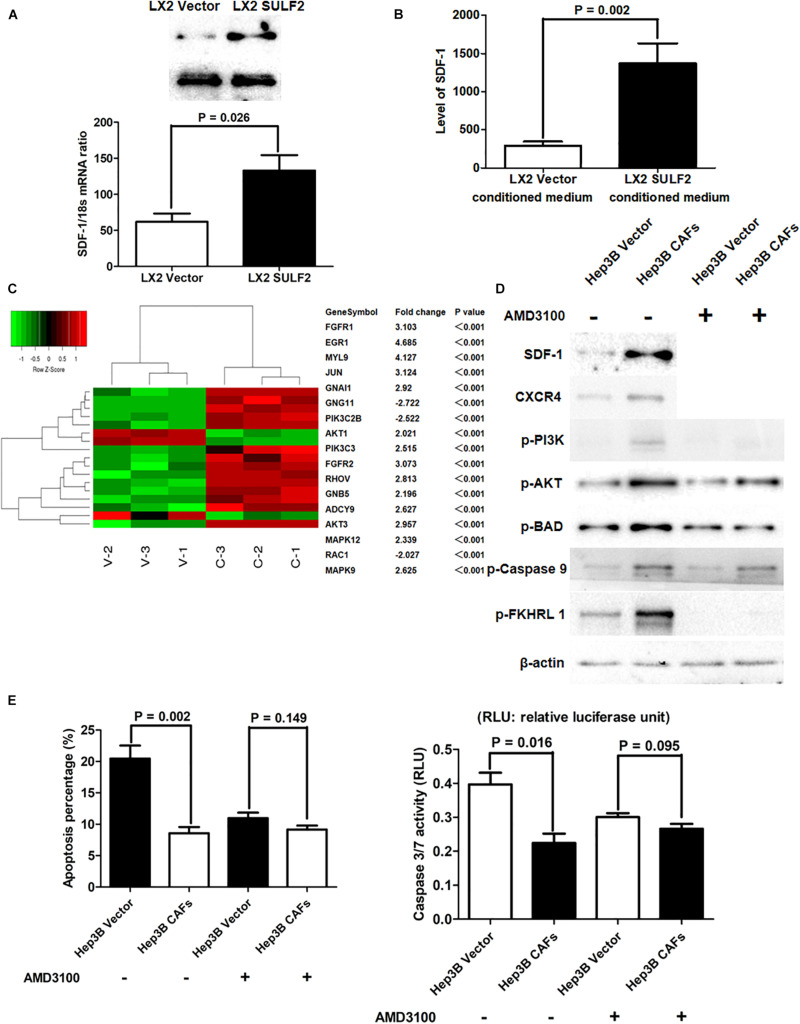
CAFs activated SDF-1/CXCR4/PI3K/AKT pathway and consequently inhibited HCC cell apoptosis. **(A)** Both RT-PCR and western immunoblotting assays showed that LX2 SULF2 cells expressed significantly more SDF-1 than LX2 Vector cells. Cell fractions: LX2 cells. **(B)** It was found by ELISA assay that there was more SDF-1 protein in conditioned medium from LX2 SULF2 cells than that from LX2 Vector cells (the protein was extracted from total cells). **(C)** Microarray profiling of mRNA expression showed that most downstream genes of SDF-1/CXCR4 signaling were remarkably upregulated in Hep3B CAFs cells in contrast to Hep3B Vector cells. **(D)** Co-culture with CAFs was found by western immunoblotting to increase expression of SDF-1, CXCR4, p-PI3K, and p-AKT in Hep3B cells, while treatment of CXCR4 inhibitor (AMD3100) did not alter the expression of phosphorylation of PI3K, AKT, BAD, caspase 9, and FKHRL 1 in Hep3B cells. Cell fractions: Hep 3B cells (the protein was extracted from total cells). **(E)** By both DAPI staining and caspase 3/7 activity assay, it was found that co-culture with CAFs suppressed cell apoptosis, whereas AMD3100 treatment did not influence Hep3B cell apoptosis apparently. CAF, carcinoma-associated fibroblast; HCC, hepatocellular carcinoma; SULF2, sulfatase 2.

The qRT-PCR and western blotting analyses revealed that the expression of SDF-1 in the SULF2-transfected LX2 cells was higher than that in the vector-transfected LX2 cells (Figure 4A). The results of ELISA revealed that the protein expression level of SDF-1 in the conditioned medium of SULF2-transfected LX2 cells was higher than that in the conditioned medium of vector-transfected LX2 cells (Figure 4B). The Hep3B cells were co-cultured with SULF2-transfected or vector-transfected LX2 cells and subjected to microarray analysis to examine the expression of downstream genes of the SDF-1/CXCR4 signaling pathway. As shown in Figure 4C, most downstream genes of the SDF-1/CXCR4 signaling pathway in the Hep3B cells co-cultured with SULF2-transfected LX2 cells were markedly upregulated than those in the Hep3B cells co-cultured with vector-transfected LX2 cells. Moreover, western blotting analysis revealed that co-culturing Hep3B cells with LX2 cells upregulated the protein expression levels of SDF-1, CXCR4, p-PI3K, and p-AKT (Figure 4D). DAPI staining and caspase 3/7 activity assay revealed that the apoptosis of Hep3B cells was inhibited when they were co-cultured with LX2 cells (Figure 4E). Additionally, the phosphorylation of pro-apoptotic proteins, including BAD, CASP9, and FKHRL 1, was upregulated in the Hep3B cells co-cultured with LX2 cells (Figure 4D). Similarly, the apoptosis of PLC/PRF/5 cells was suppressed when they were co-cultured with CAFs cells (Supplementary Figure 3A). Additionally, the expression levels of SDF-1, CXCR4, p-PI3K, and p-AKT and the levels of phosphorylated pro-apoptotic proteins, including BAD, CASP9, and FKHRL 1, were upregulated in the PLC/PRF/5 cells co-cultured with LX2 cells (Supplementary Figure 3B). However, treatment with AMD3100, an inhibitor of CXCR4, did not repress apoptosis (Figure 4E) but promoted the phosphorylation of PI3K, AKT, BAD, CASP9, and FKHRL 1 in the Hep3B cells co-cultured with LX2 (Figure 4D). This indicated that the SDF-1/CXCR4 cascade was crucial for CAF-mediated inhibition of HCC cell apoptosis.

### Sulfatase 2-Induced Carcinoma-Associated Fibroblasts Promoted Epithelial-to-Mesenchymal Transition of Hepatocellular Carcinoma Cells by Upregulating SNAI1 and Activating the SDF-1/CXCR4 Signaling Pathway

**FIGURE 5:**
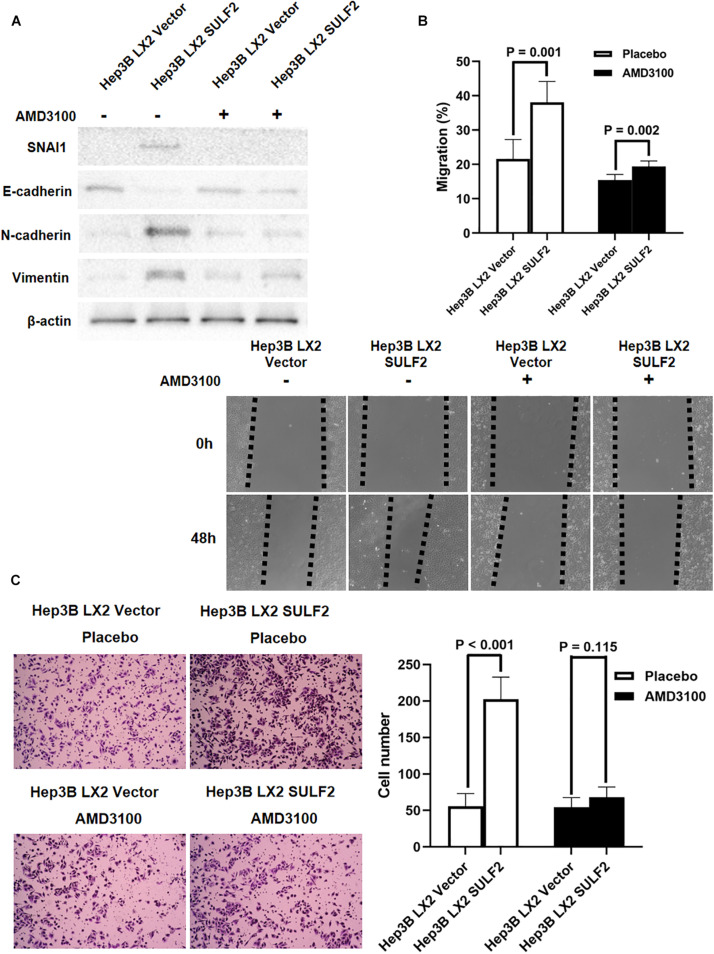
CAFs driven by SULF2 induced HCC EMT phenotype via upregulating SNAI1 by activating SDF-1/CXCR4 signaling. **(A)** By western immunoblotting assay, it was found that Hep3B LX2 SULF2 cells had significantly more expression of SNAI1, N-cadherin, and Vimentin and less E-cadherin expression than Hep3B LX2 Vector cells, while co-culture with LX2 SULF2 cells did not alter the expression of E-cadherin, SNAI1, N-cadherin, and Vimentin after treatment of AMD3100. Cell fractions: Hep 3B cells (the protein was extracted from total cells). **(B)** As assessed by wound healing assay, the migration ability of Hep3B cells was found to be accelerated by co-culture with LX2 SULF2 cells, which was weakened by treatment of AMD3100. **(C)** Transwell invasion assay showed that co-culture with LX2 SULF2 cells consolidated the invasion capacity of Hep3B cells and AMD3100 treatment attenuated the regulatory effect of co-culture with LX2 SULF2 cells on the Hep3B cell invasion ability. CAF, carcinoma-associated fibroblast; SULF2, sulfatase 2; HCC, hepatocellular carcinoma; EMT, epithelial-to-mesenchymal transition.

Recent studies have reported that CAFs promote cancer invasion and metastasis and induce EMT (Yamaguchi et al., 2018). The role of SULF2-activated CAFs in inducing EMT of neighboring HCC cells was examined. The SULF2-transfected or vector-transfected LX2 cells were co-cultured with Hep3B cells for 72 h. Western blotting revealed that compared with those in the Hep3B cells co-cultured with vector-transfected LX2 cells, the expression levels of SNAI1, CDH2, and VIM were upregulated, and the expression level of CDH1 was downregulated in the Hep3B cells co-cultured with SULF2-transfected LX2 cells (Figure 5A). The results of the wound-healing assay revealed that the Hep3B cells co-cultured with SULF2-transfected LX2 cells exhibited enhanced migration (Figure 5B). The invasion ability of Hep3B cells was assessed using Transwell chamber coated with Matrigel. The Hep3B cells co-cultured with SULF2-transfected LX2 cells exhibited enhanced invasion (Figure 5C). Treatment with AMD3100 mitigated the downregulated expression of CDH1 and the upregulated expression of SNAI1, CDH2, and VIM in the Hep3B cells co-cultured with SULF2-transfected LX2 cells (Figure 5A). Additionally, treatment with AMD3100 mitigated the enhanced migration and invasion of Hep3B cells co-cultured with SULF2-transfected LX2 cells (Figures 5B,C).

### SDF-1/CXCR4 Axis-Mediated miR-153-3p Downregulation Promoted Epithelial-to-Mesenchymal Transition of Hepatocellular Carcinoma Cells by Targeting SNAI1

**FIGURE 6:**
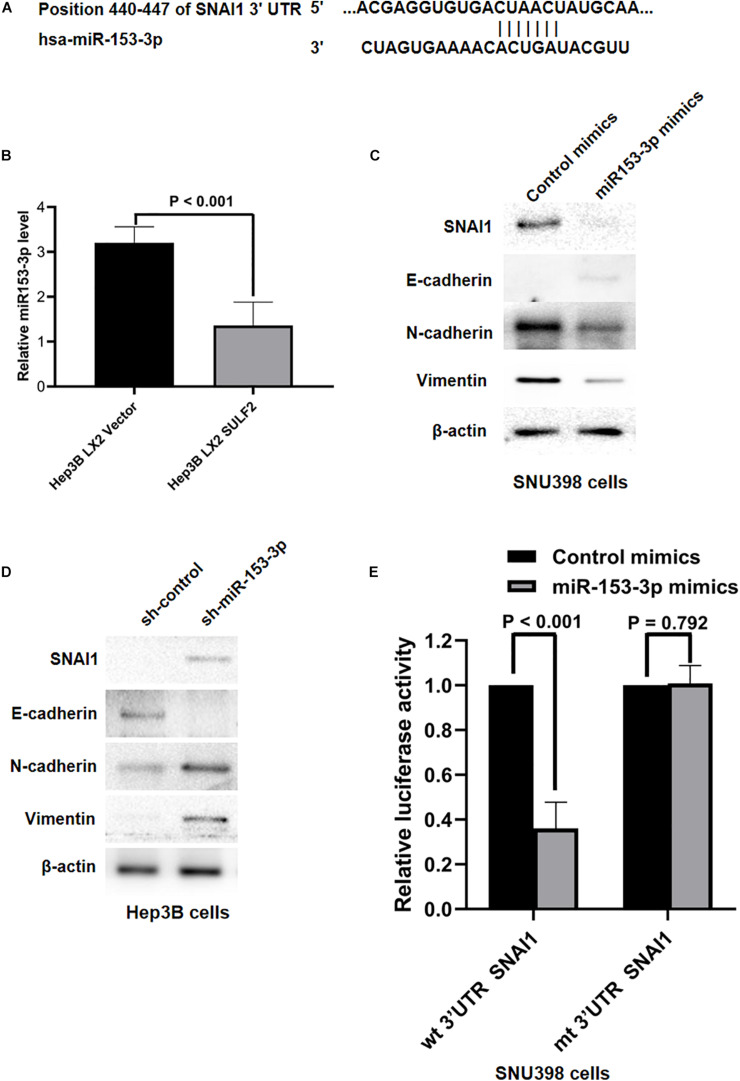
SDF-1/CXCR4 axis inhibited miR-153-3p expression and then induced EMT of HCC cells via upregulating SNAI1. **(A)** The data from TargetScan 7.2 database showed the positions 440–447 of SNAI1 3′-UTR had the complementary sequence of miR-153-3p. **(B)** As assessed by qRT-PCR, it was found that Hep3B LX2 SULF2 cells had significantly less miR-153-3p in contrast to Hep3B LX2 Vector cells. **(C)** Western immunoblotting showed that enhanced expression of miR-153-3p in SNU398 cells decreased expression of SNAI1, N-cadherin, and Vimentin and upregulated E-cadherin magnificently. Cell fractions: SNU398 cells (the protein was extracted from total cells). **(D)** Knockdown of miR-153-3p with miRNA inhibitors in Hep3B cells resulted in upregulation of SNAI1, N-cadherin, and Vimentin, while it led to loss of E-cadherin. Cell fractions: Hep 3B cells (the protein was extracted from total cells). **(E)** Luciferase reporter assay demonstrated that miR-153-3p overexpression by mimics treatment markedly decreased the luciferase activity of plasmid containing wt 3′-UTR of SNAI1 rather than mt 3′-UTR of SNAI1 in SNU398 cells. EMT, epithelial-to-mesenchymal transition; HCC, hepatocellular carcinoma; SULF2, sulfatase 2.

Various studies have reported that miRNAs were associated with the initiation of EMT of cancer cells and HCC progression (Roy et al., 2018; Tan et al., 2018; Wang et al., 2018; Liu et al., 2019). The role of miRNAs in the SDF-1/CXCR4 signaling-mediated regulation of SNAI1 expression was examined in the HCC cells. The miRNAs that target *SNAI1* were identified using TargetScan 7.2. The complementary sequence of miR-153-3p was detected at positions 440–447 of *SNAI1* 3′-untranslated region (UTR) (Figure 6A), which indicated that miR-153-3p can potentially repress *SNAI1* mRNA expression. The levels of miR-153-3p in the Hep3B cells co-cultured with SULF2-transfected LX2 cells or vector-transfected LX2 cells were examined using qRT-PCR. Compared with those in the Hep3B cells co-cultured with vector-transfected LX2 cells, the miR-153-3p expression levels were downregulated in the Hep3B co-cultured with SULF2-transfected LX2 cells (Figure 6B). To confirm the ability of miR-153-3p to repress *SNAI1* expression in the HCC cells, hsa-miR-153-3p mimics (GenePharma Co., Shanghai, China) were used to overexpress miR-153-3p in the SNU398 cells, following the manufacturer’s instructions. The qRT-PCR analysis revealed that the expression of miR-153-3p was upregulated in the hsa-miR-153-3p mimic-transfected SNU398 cells (Supplementary Figure 4A). As shown in Figure 6C, the overexpression of miR-153-3p downregulated the expression of SNAI1, CDH2, and VIM and upregulated the expression of CDH1 in the SNU398 cells. Conversely, transfection with miR-153-3p inhibitors (Supplementary Figure 4B) upregulated the expression of SNAI1, CDH2, and VIM (Figure 6D) and downregulated the expression of CDH1 in the Hep3B cells. Furthermore, the results of the luciferase reporter assay demonstrated that the luciferase activity of the construct containing wild-type 3′-UTR of *SNAI1* was lower than that of the construct containing mutant 3′-UTR of *SNAI1* in the miR-153-3p mimic-transfected SNU398 cells (Figure 6E). Interestingly, the overexpression of miR-153-3p suppressed the migration and invasion of SNU398 cells. In contrast, the silencing of miR-153-3p facilitated the migration and invasion of SNU398 cells (Supplementary Figures 5A,B). These findings indicated that the SDF-1/CXCR4 signaling pathway inhibited miR-153-3p and consequently promoted EMT of HCC cells by upregulating SNAI1, which is mediated by the direct binding of SDF-1 to *SNAI1* 3′-UTR.

### SDF-1/CXCR4 Signaling-Induced Non-coding RNA OIP5-AS1 Functioned as a Competing Endogenous RNA to Modulate SNAI1 Expression by Sponging miR-153-3p

**FIGURE 7:**
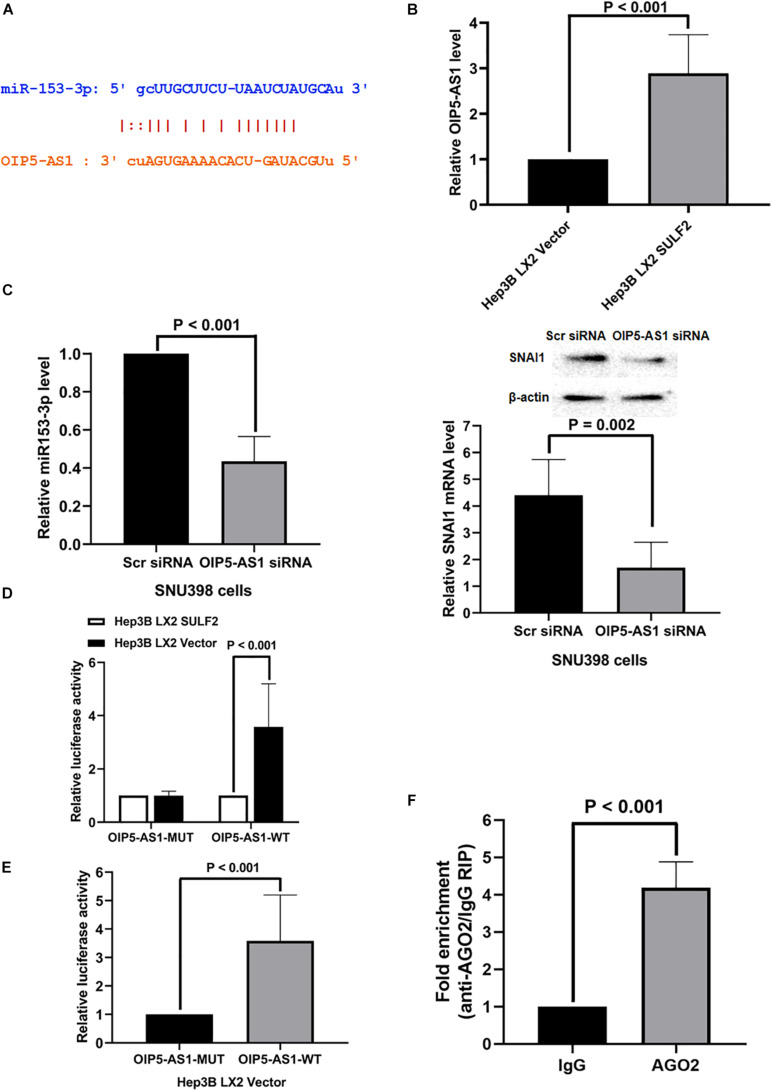
OIP5-AS1 mediated by SDF-1/CXCR4 axis functioned as a ceRNA modulating SNAI1 expression via sponging miR-153-3p. **(A)** The miRCode database showed that there was one conservative binding site of OIP5-AS1 with miR-153-3p. **(B)** qRT-PCR assay displayed that Hep3B LX2 SULF2 cells had significantly more OIP5-AS1 expression than Hep3B LX2 Vector cells. **(C)** Knockdown of OIP5-AS1 was found to enhance miR-153-3p expression (qRT-PCR) and to decrease SNAI1 expression (qRT-PCR and western immunoblotting) (the protein was extracted from total cells). **(D)** Luciferase reporter assay displayed that there was a higher luciferase activity of OIP5-AS1-WT in Hep3B LX2 Vector cells than Hep3B LX2 SULF2 cells, but there was no significant change detected of luciferase activity of OIP5-AS1-MUT plasmid between Hep3B LX2 Vector cells and Hep3B LX2 SULF2 cells. **(E)** It was shown by luciferase reporter assay that the luciferase activity detected in Hep3B LX2 Vector cells with OIP5-AS1-WT plasmid was dramatically higher than that with OIP5-AS1-MUT plasmid. **(F)** RNA immunoprecipitation (RIP) displayed that OIP5-AS1 was significantly enriched by the AGO2 antibody. ceRNA, competing endogenous RNA.

Non-coding RNAs (ncRNAs) are reported to function as a ceRNA to sponge miRNAs (Liu et al., 2019; Liang et al., 2020). The key ceRNAs that regulate miR-153-3p expression were examined. miRCode was used to search the potential ncRNAs that can bind miR-153-3p. NcRNA OIP5-AS1 was predicted to bind to miR-153-3p. As shown in Figure 7A, one conserved binding site of OIP5-AS1 was identified in miR-153-3p. The analysis of TCGA database revealed that the expression of OIP5-AS1 in the HCC tissues was 1.28-fold higher than that in the adjacent non-tumor tissues (*P* = 0.0026). Consistently, the qRT-PCR analysis of OIP5-AS1 in 102 HCC samples revealed that the expression of OIP5-AS1 in the HCC tissues was significantly higher than that in the adjacent liver tissues (Supplementary Figure 6B). The role of OIP5-AS1 in SDF-1/CXCR4 signaling-induced modulation of SNAI1 in the Hep3B cells co-cultured with SULF2-transfected LX2 cells was examined. The level of OIP5-AS1 in the Hep3B cells co-cultured with SULF2-transfected or vector-transfected LX2 cells was examined using qRT-PCR. As shown in Figure 7B, the expression of OIP5-AS1 in the Hep3B cells co-cultured with SULF2-transfected LX2 cells was higher than that in the Hep3B cells co-cultured with vector-transfected LX2 cells. The siRNA-mediated knockdown of OIP5-AS1 upregulated miR-153-3p and downregulated mRNA and proteins levels of SNAI1 in the SNU398 cells (Figure 7C). These findings indicated that the activation of the SDF-1/CXCR4 pathway promoted OIP5-AS1 expression in the HCC cells and that OIP5-AS1 expression level was positively correlated with the expression of both miR-153-3p and SNAI1. Next, the direct interaction between miR-153-3p and OIP5-AS1 was examined. The OIP5-AS1 luciferase reporter plasmids with wild-type miR-153-3p binding sites (OIP5-AS1-WT) or mutant miR-153-3p binding sites (OIP5-AS1-MUT) were constructed. The expression of miR-153-3p in the Hep3B cells co-cultured with vector-transfected LX2 cells (Figure 6B) was higher than that in the Hep3B cells co-cultured with SULF2-transfected LX2 cells. Hence, the Hep3B cells co-cultured with vector-transfected or SULF2-transfected LX2 cells were transfected with OIP5-AS1-WT or OIP5-AS1-MUT plasmids. The luciferase activity of OIP5-AS1-WT in the Hep3B cells co-cultured with vector-transfected LX2 cells was higher than that in the Hep3B cells co-cultured with SULF2-transfected LX2 cells. In contrast, the luciferase activity of OIP5-AS1-MUT was not significantly different between the Hep3B cells co-cultured with vector-transfected LX2 cells and those co-cultured with SULF2-transfected LX2 cells (Figure 7D). Additionally, the luciferase activity of OIP5-AS1-WT was markedly higher than that of OIP5-AS1-MUT in the Hep3B cells co-cultured with vector-transfected LX2 cells (Figure 7E). Next, we examined whether OIP5-AS1 functioned as a ceRNA in the HCC cells through the RNA-induced silencing complex catalytic subunit Argonaute 2 (AGO2). The results of the RIP assay revealed that OIP5-AS1 significantly immunoprecipitated with the anti-AGO2 antibody (Figure 7F; *P* < 0.05). These results suggested that OIP5-AS1 functioned as a molecular sponge for miR-153-3p in HCC cells.

### OIP5-AS1 Promoted Hepatocellular Carcinoma Xenograft Growth and Induced Epithelial-to-Mesenchymal Transition of Hepatocellular Carcinoma Cells by Upregulating SNAI1 Expression *in vivo*

**FIGURE 8:**
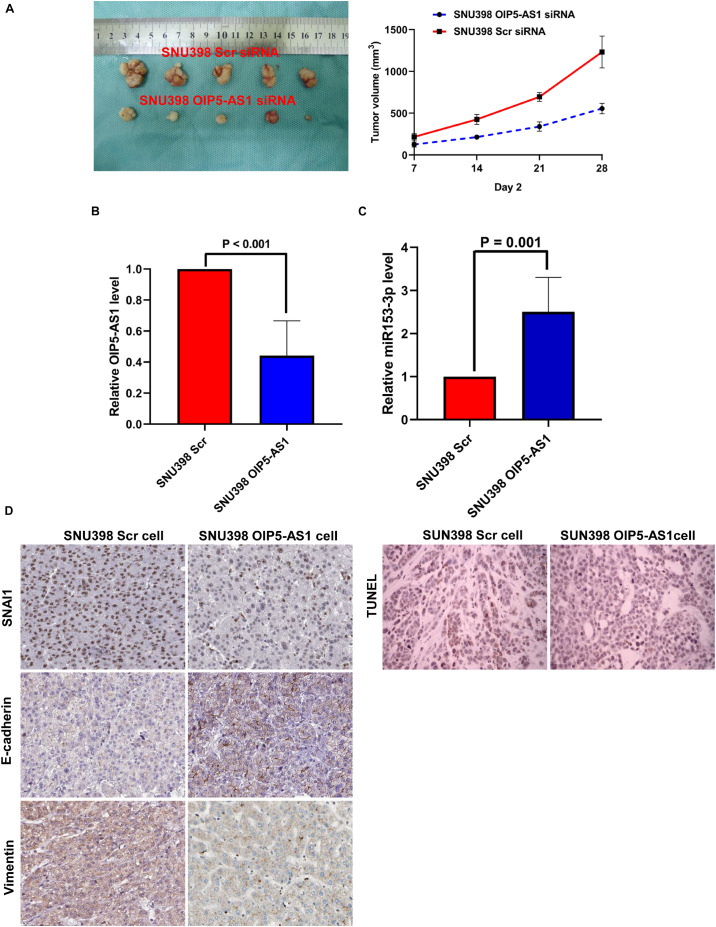
OIP5-AS1 accelerated the growth of HCC *in vivo* and induced EMT phenotype of HCC cells via upregulating SNAI1 expression. **(A)** By measuring tumor growth curves and tumor weight, it was found that knockdown of OIP5-AS1 attenuated the growth of HCC xenografts. **(B)** qRT-PCR analysis showed that SNU398 OIP5-AS1 cells had significantly less OIP5-AS1 expression than SNU398 Scr cells. **(C)** SNU398 OIP5-AS1 cells were found by qRT-PCR assay to express more miR-153-3p expression than SNU398 Scr cells. **(D)** IHC staining was carried out in HCC xenograft tissues, and it was found that there was magnificently lower level of both SNAI1 and Vimentin, and more E-cadherin is detected in SNU398 OIP5-AS1 cells than SNU398 Scr cells. HCC, hepatocellular carcinoma; EMT, epithelial-to-mesenchymal transition; IHC, immunohistochemical.

The role of OIP5-AS1 in EMT of HCC and the underlying mechanism were examined. After our pre-experimental verification, we found that SNU398 cells exhibit high expression of OIP5-AS1 and had a better effect on tumor formation *in vivo*, so SNU398 cells were selected for the next experiments. The si-OIP5-AS1-transfected SNU398 cells or si-Scr siRNA-transfected SNU398 cells were subcutaneously implanted into nude mice. The analysis of tumor growth curves and tumor weight revealed that the growth of HCC xenografts was mitigated in the SNU398 OIP5-AS1 siRNA group (Figure 8A). HCC xenografts were harvested from both groups and subjected to qRT-PCR analysis. Compared with those in the SNU398 Scr siRNA group, the expression levels of OIP5-AS1 were downregulated and the level of miR-153-3p was upregulated in the SNU398 OIP5-AS1 siRNA group (Figures 8B,C). IHC analysis of HCC xenograft tissues revealed that compared with the SNU398 Scr siRNA group, the SNU398 OIP5-AS1 siRNA group exhibited downregulated levels of SNAI1 and VIM and upregulated levels of CDH1 (Figure 8D). Also, TUNEL assay showed that the SNU398 OIP5-AS1 siRNA group exhibited upregulated levels of apoptosis rate when compared with the SNU398 Scr siRNA group (Figure 8D). The nuclear immunostaining of histone H3 was as the positive control. These results suggested that OIP5-AS1 promoted SNAI1 expression by repressing miR-153-3p and consequently induced EMT of HCC cells and promoted tumor growth *in vivo*.

## Discussion

The tumor microenvironment, which has well-regulated intercellular communications, promotes HCC progression and metastases by sustaining the cell proliferation pathway, evading growth suppressors, maintaining replicative immortality, initiating angiogenesis, promoting invasion and metastasis, regulating energy metabolism, and eluding cancer immune surveillance (Zhang et al., 2017; Cheng et al., 2018; Cho et al., 2018; Bu et al., 2019). CAFs, which are the most common type of stroma cells in the HCC microenvironment, mediate the tumor–stromal crosstalk. Various studies have reported that CAFs promote HCC progression by enhancing the proliferation, migration, and invasion of the tumor cells (Liu et al., 2016; Fang et al., 2018). However, the interaction between HCC cells and CAFs has not been completely elucidated. SULF2, a member of 17 human sulfatase genes, regulates the interactions of HSPGs with extracellular signaling proteins by removing the intra-polymer sulfate groups from the 6-*O* position of HSPGs. Microarray analysis revealed that SULF2 was upregulated in 79 of the 139 (57%) primary HCCs and that patients with upregulated SULF2 expression exhibited poor prognosis and an increased rate of relapse postsurgery (Lai et al., 2008). However, the role of SULF2 in CAF differentiation and maintenance has not been elucidated. In this study, the analysis of TCGA database revealed that the mRNA expression level of *SULF2* in the HCC tissues was significantly and positively correlated with that of CAF biomarkers, including *ACTA2*, *FAP*, and *POSTN*. This indicated that SULF2 was positively correlated with CAF differentiation in the HCC microenvironment. Furthermore, SULF2 and CAF biomarkers were associated with unfavorable prognosis in patients with HCC. These findings were validated using IHC analysis of 102 HCC samples. CAFs in the cancer microenvironment are derived from the activation of fibroblasts (Fukino et al., 2004), EMT of epithelial cells (William Petersen et al., 2001; Peran et al., 2020), EMT of endothelial cells (Zeisberg et al., 2007; Xu et al., 2021), and differentiation of bone marrow mesenchymal cells (Quante et al., 2011; Raz et al., 2018). LX2 cells used in this research are spontaneously immortalized stellate cells, while CAFs represent a very heterogeneous cell population with different origins, so we verified the feature of stellate cells of LX2 cells compared with primary HSCs (Supplementary Figure 8A). The protein expression of stellate cell markers (α-SMA and Col 1) was upregulated in both LX2 cells and primary HSCs. In order to verify the function of SULF2, we established SULF2 overexpressing LO2 clones and then co-cultured with LX2 cells. We detected the mRNA expression of CAFs’ markers (α-SMA, FAP, and POSTEN), which were upregulated in LX2 cells co-cultured with SULF2 overexpressing LO2 cells (Supplementary Figure 8). However, the source of CAFs in the HCC microenvironment is unclear. Hence, this study examined the role of SULF2 secreted by HCC cells in the differentiation of HSCs into CAFs. There is heterogeneity in different cell lines, so in order to eliminate the interference of heterogeneity, we used high- and low-SULF2-expressing cells (Hep3B, PLC/PRF/5, and SNU398) to verify the experimental results. Naïve cells in HCC may play an important and novel role, and what effect of conditioned medium of CAFs on naïve HCC cell lines remains unknown. We did not involve related research, which is regretful. In the following research, we will explore the relationship among CAF, HCC, and naïve cells. Co-culturing LX2 cells with SNU398, which exhibited upregulated expression levels of SULF2, increased the expression levels of CAF biomarkers (ACTA2, FAP, and POSTN). This finding was further verified by co-culturing the LX2 cells with SULF2-transfected Hep3B cells. The mechanistic investigation revealed that SULF2 activated the TGFβ1/SMAD3 signaling pathway and consequently upregulated the expression of both ACTA2 and POSTN by binding to the promoter of *SMAD3*. A similar mechanism was reported in the lung fibroblast model in which TGFβ1 treatment induced myofibroblast-like morphological changes and upregulated ACTA2 protein expression (Evans et al., 2003; Morrone et al., 2020). These data suggested that SULF2 secreted by the HCC cells promoted the differentiation of HSCs into CAFs through the TGFβ1/SMAD3 signaling pathway.

Luo et al. (2018) and Ju et al. (2009) reported that activated HSCs were positively correlated with unfavorable prognosis after curative resection. Previously, we had demonstrated that co-culturing Huh7 cells with TIMP-1-induced CAFs activated the SDF-1/CXCR4/PI3K/AKT signaling pathway and consequently suppressed cell apoptosis (Song et al., 2015). In this study, SULF2 secreted from the HCC cells promoted the differentiation of HSCs into CAFs, which inhibited HCC cell apoptosis through the secretion of SDF-1 and the activation of the SDF-1/CXCR4/PI3K/AKT pathway. This indicated that SULF2-induced CAFs and TIMP-1-induced CAFs exhibit similar characteristics in the HCC microenvironment. Similarly, we observed the proliferation status of HCC in co-culture systems and found that compared with Hep 3B LX2 Vector, the proliferation increased when Hep 3B was co-cultured with LX2 SULF2. Also, the apoptosis status of CAFs after co-cultured had no change. This indicated that co-culture with LX2 SULF2 could promote the proliferation of HCC while it had no effect on the apoptosis rate of CAFs (Supplementary Figures 8F,G).

Recent studies have reported that long ncRNAs (lncRNAs) and miRNAs, which are dysregulated in HCC, play a critical role in hepatocarcinogenesis (Zou et al., 2016; Wong et al., 2018). LncRNAs, a type of ncRNAs, are defined as RNAs with more than 200 nucleotides. Additionally, lncRNAs undergo processing, including splicing, capping, polyadenylation, and editing, but lack open reading frames. Furthermore, lncRNAs are reported to regulate HCC pathogenesis by modulating the expression and activity of miRNAs, mRNAs, and proteins (Li et al., 2017; Xiong et al., 2017; Huang Y. et al., 2018; Lim et al., 2019). The understanding of lncRNA function and mechanism will provide novel insights for the diagnosis and treatment of HCC. Previous studies have reported that 74 types of lncRNAs are aberrantly expressed in HCC, but the underlying mechanism has not been elucidated (Kim et al., 2019). In this study, the expression of OIP5-AS1 in the HCC tissues was upregulated when compared with that in the adjacent liver tissues in both TCGA database and the HCC clinical specimens. *In vitro* experiments revealed that the hyperactivation of the SDF-1/CXCR4 signaling pathway induced by neighboring CAFs upregulated OIP5-AS1 expression in the HCC cells. Several studies have demonstrated that lncRNAs maintain the stability and translation of mRNA by functioning as a ceRNA to sponge miRNAs and consequently regulate the downstream cell signaling in HCC (Yuan et al., 2014; Wang et al., 2017; Huang X. et al., 2018; Wang Y.G. et al., 2019). The function and mechanism of miR-153-3p in HCC progression have not been previously reported. *In vitro* studies demonstrated that CAFs upregulated SNAI1 expression in the neighboring HCC cells by regulating the SDF-1/CXCR4 signaling pathway. The mechanistic investigation revealed that miR-153-3p directly repressed *SNAI1* expression in the HCC cells, which reversed the EMT phenotype of HCC cells. Furthermore, bioinformatics analysis and *in vitro* experiments revealed that OIP5-AS1 functions as a ceRNA to sponge miR-153-3p in the HCC cells. Finally, *in vivo* experiments confirmed that the overexpression of OIP5-AS1 promoted the growth of HCC xenografts, induced EMT of the HCC cells, and upregulated SNAI1 expression. These data indicated that CAFs derived from SULF2 secreted by HCC cells increased OIP5-AS1 expression through the SDF-1/CXCR4 signaling pathway. OIP5-AS1 upregulated *SNAI1* expression by sponging miR-153-3p and consequently promoted HCC progression.

In our previous related research, it has been confirmed that the expression of SULF2 is low in Hep 3B and high in huh7 and SNU398 cells (Lai et al., 2010a, b; Zheng et al., 2013). In this study, we detected the CAF markers in LX2 cells and CAFs cells, while we did not detect the expression of CAF markers in HCC cells. In the next study, it is meaningful to study whether HCC cells express CAF markers and how they influence HCC cells. Various studies have reported that inhibiting the expression of SULF2 can downregulate the expression of verification indicators, such as NFκB, TNF-α, IL-1β, IL-4, and IL-6 (Alyoussef and Al-Gayyar, 2018). Previous studies have demonstrated the correlation between SULF2 and the occurrence of liver fibrosis. The knockout of *SULF2* significantly inhibited the occurrence of liver fibrosis (Kim et al., 2020). As inflammation, liver fibrosis, and HCC are correlated, SULF2 may play an important role in these processes. Further studies are needed to elucidate the mechanism of SULF2 in inflammation and liver fibrosis.

In this study, SULF2 promoted the differentiation of LX2 cells into CAFs, which promoted the progression of HCC through the SDF-1/CXCR4/PI3K/AKT pathway and OIP5-AS1/miR-153-3p/SNAI1 axis. Further studies are needed to elucidate the mechanism of SULF2 in HCC. Future studies must examine the role of SULF2 in hepatitis, liver cirrhosis, and liver cancer. Moreover, the immune cells in the tumor microenvironment may play an important role in the occurrence and development of liver cancer. Follow-up studies are needed to examine the role of CAFs in the immune system and liver cancer.

## Conclusion

**FIGURE 9:**
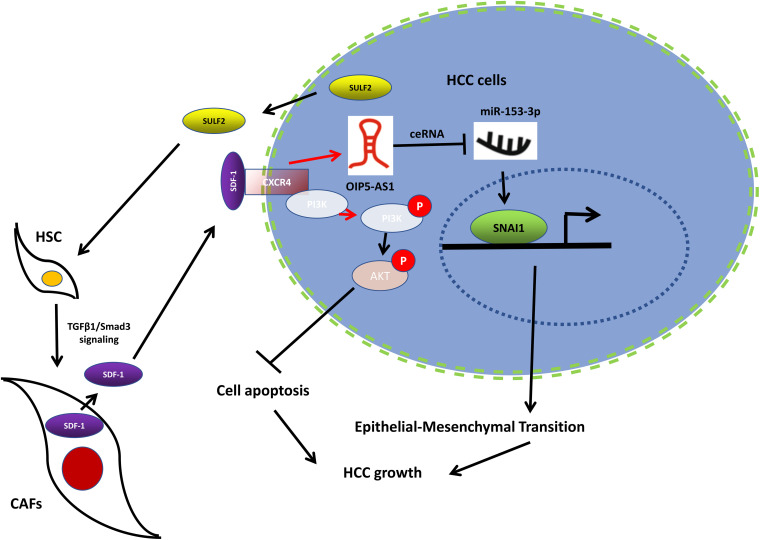
Working model of the mechanism by which CAFs activated by SULF2 in HCC microenvironment promoted tumor growth. CAF, carcinoma-associated fibroblast; SULF2, sulfatase 2; HCC, hepatocellular carcinoma.

As shown in Figure 9, the findings of this study indicated that SULF2 secreted by the HCC cells promoted the differentiation of HSCs into CAFs through the TGFβ1/SMAD3 signaling pathway. SULF2-induced CAFs inhibited HCC apoptosis by activating the SDF-1/CXCR4/PI3K/AKT signaling pathway. Additionally, OIP5-AS1, which was aberrantly upregulated in the HCC tissues, sponged miR-153-3p. This resulted in the repression of SNAI1 expression, which promoted HCC progression through the induction of EMT. This study elucidated a novel mechanism involved in the crosstalk between HCC cells and CAFs in the tumor microenvironment, which can aid in the development of novel and efficient therapeutic strategies for primary liver cancer.

## Data Availability Statement

The datasets presented in this study can be found in online repositories. The names of the repository/repositories and accession number(s) can be found in the article/Supplementary Material.

## Ethics Statement

The studies involving human participants were reviewed and approved by the Ethical Review of Research Involving Human Subjects of the First Hospital of Xian Jiaotong University. The patients/participants provided their written informed consent to participate in this study. The animal study was reviewed and approved by First Affiliated Hospital of Xi’an Jiaotong University.

## Author Contributions

XZ and QL designed the whole project and supervised all the experiments. CW and CS performed the molecular biology experiment and analysis. XG and TS performed the clinical sample collection. SH performed all the animal experiments. All authors have read and approved the final manuscript.

## Conflict of Interest

The authors declare that the research was conducted in the absence of any commercial or financial relationships that could be construed as a potential conflict of interest.

## Abbreviations

HCC: hepatocellular carcinoma
HSC: hepatic stellate cells
CAFs: carcinoma-associated fibroblasts
EMT: epithelial-to-mesenchymal transition
TCGA: The Cancer Genome Atlas
SULF2: sulfatase 2
lncRNA: long non-coding RNA
ceRNA: competing endogenous RNA
TGFβ1: transforming growth factor-β1
SDF-1: stromal cell-derived factor 1
CXCR4: C-X-C motif chemokine receptor 4
SNAI1: snail family transcriptional repressor 1.
