# Supplementary material for: Sulfatase 2-Induced Cancer-Associated Fibroblasts Promote Hepatocellular Carcinoma Progression via Inhibition of Apoptosis and Induction of Epithelial-to-Mesenchymal Transition
Source: Front Cell Dev Biol. 2021 Apr 6;9:631931. doi: 10.3389/fcell.2021.631931 (PMC8056031; doi:10.3389/fcell.2021.631931)
Supplement: Supplementary file 1 [file Data_Sheet_1.PDF]

Supplementary Fig 1

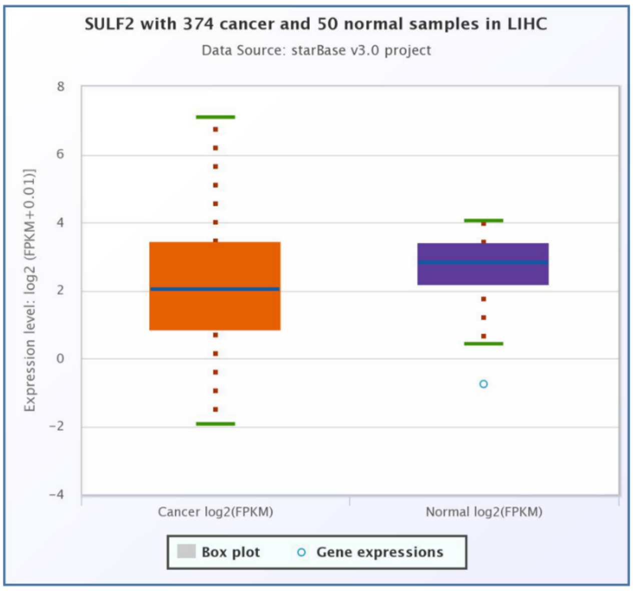

Mean cancer expression = 8.54 vs. mean normal liver exxpression = 7.58
Fold change =1.13, P = 0.026

Supplementary figure 1 Analysis of TCGA database showed that SULF2 mRNA was found up-regulated significantly in HCC tissues

Supplementary Fig 2

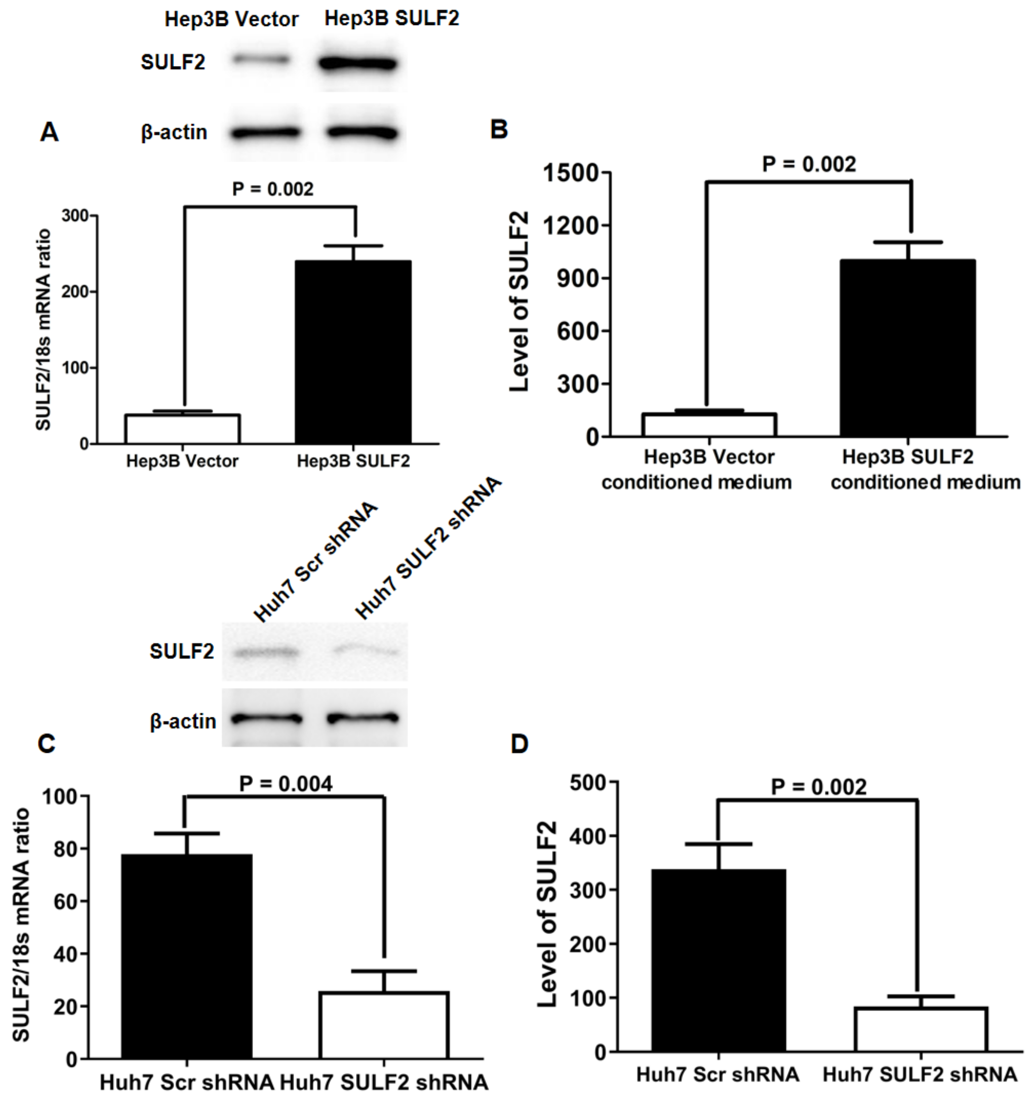

Supplementary figure 2 A Transfection with SULF2 expressing plasmid was verified by both RT-PCR and Western immunoblotting assays to increase SULF2 expression in Hep3B cells. Cell fractions: Hep 3B cells. **B** ELISA assay displayed that there was apparently more SULF2 protein in Hep3B SULF2 cell medium than Hep3B Vector cell medium. **C** The shRNA sequences against SULF2 was found to silence SULF2 expression in Huh7 cells by RT-PCR and Western immunoblotting assays. Cell fractions: Huh7 cells. **D** It was also found less SULF2 expression in medium from Huh7 SULF2

shRNA cells than those from Huh7 Scr shRNA cells by ELISA assessment.

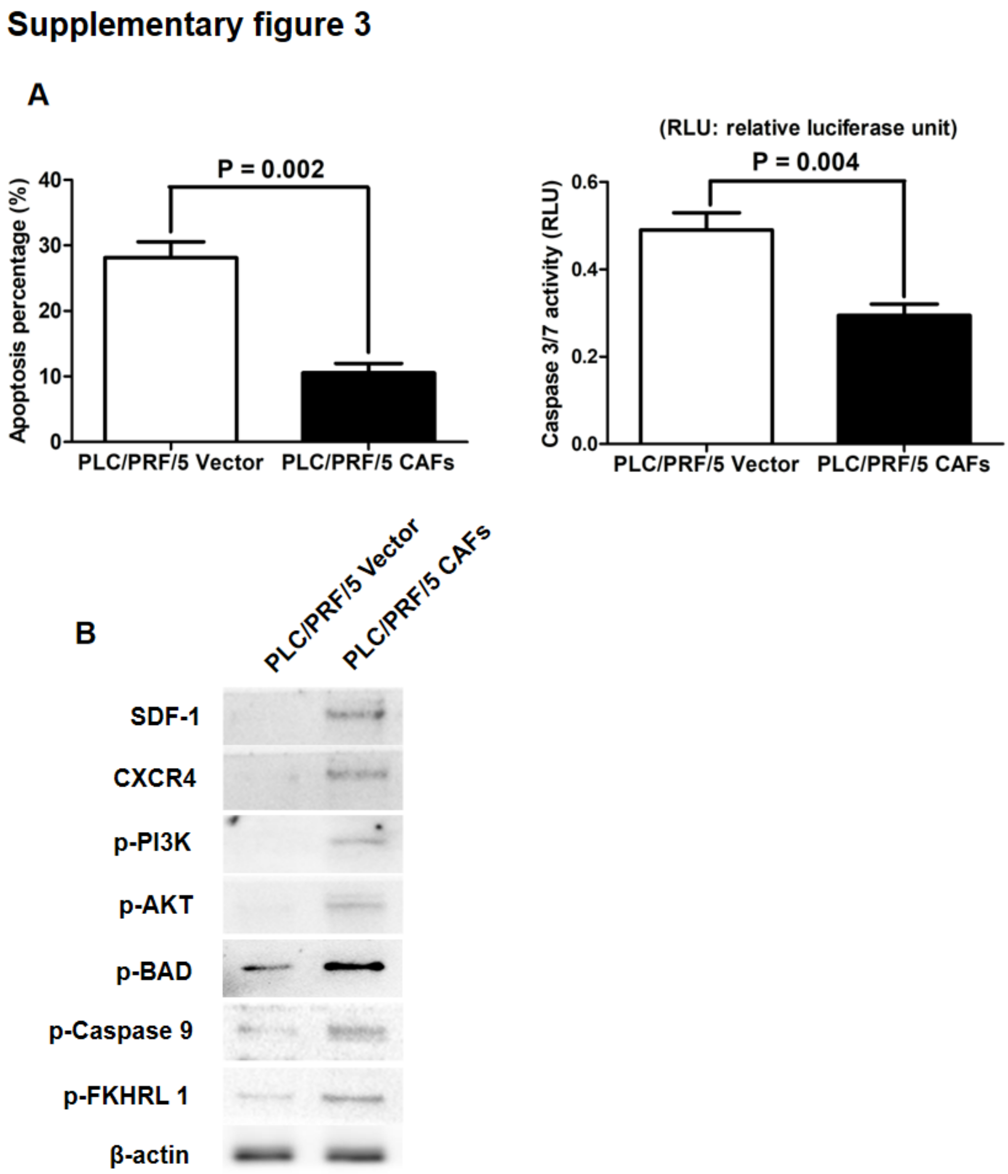

Supplementary figure 3 CAFs inhibited cell apoptosis of PLC/PRF/5 cells via inducing activating SDF-1/CXCR4/p-PI3K/p-AKT axis. **A** By both DAPI staining and Caspase 3/7 activity assay, co-culture with CAFs repressed cell apoptosis of PLC/PRF/5 cells. **B** Co-culture with CAFs was found by Western immunoblotting to increase the expression of SDF-1, CXCR4, p-PI3K, and p-AKT, while enhancing phosphorylation

of BAD、Caspase 9 and FKHRL 1 in PLC/PRF/5 cells. Cell fractions: PLC/PRF/5 cells.

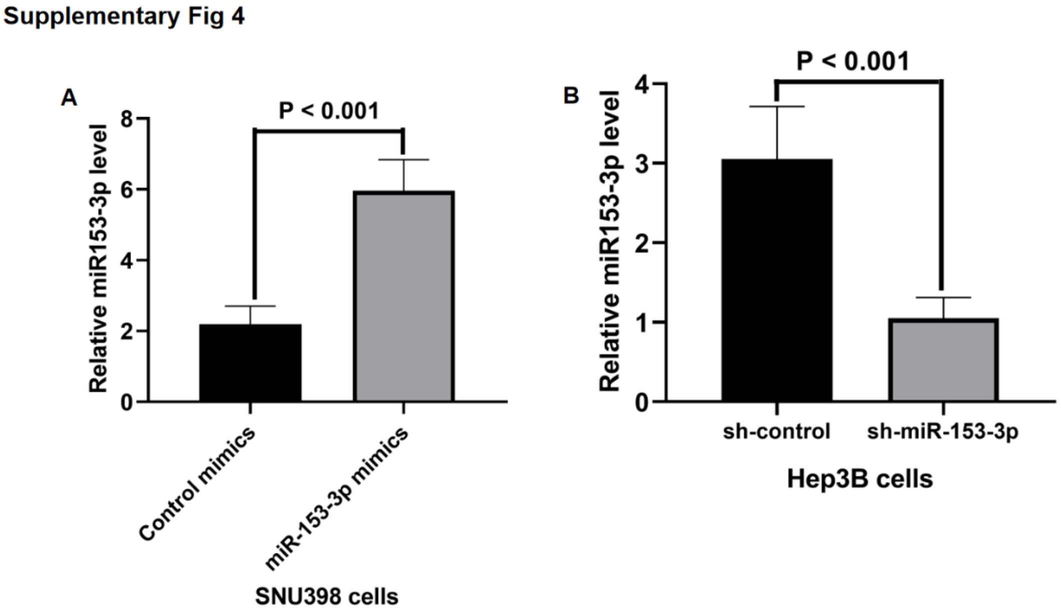

Supplementary figure 4 A qRT-PCR assessment showed that miR-153-3p expression was enhanced magnificently by transfection with hsa-miR-153-3p mimics in SNU398 cells. **B** Treatment of miRNA inhibitors against hsa-miR-153-3p decreased miR-153-3p expression in Hep3B cells dramatically.

Supplementary Fig 5

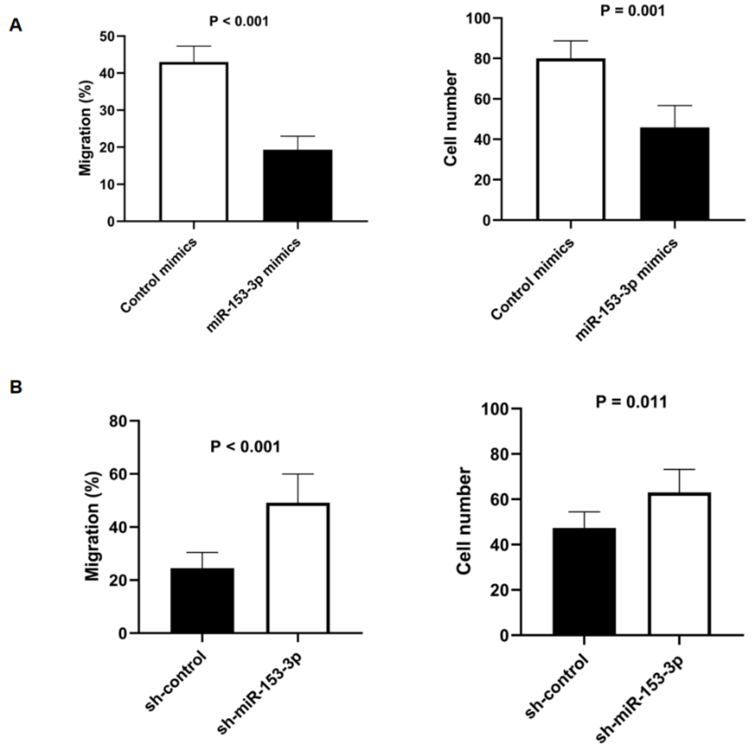

Supplementary figure 5 A By wound healing assay and transwell invasion assay, it was found that enhanced expression of miR-153-3p in SNU398 cells suppressed the capacities of migration and invasion. **B** Wound healing assay and transwell invasion assay displayed that knockdown of miR-153-3p in Hep3B cells promoted cell migration and invasion.

Supplementary Fig 6

A

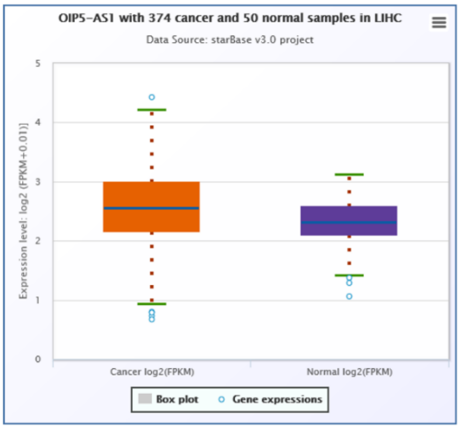

B

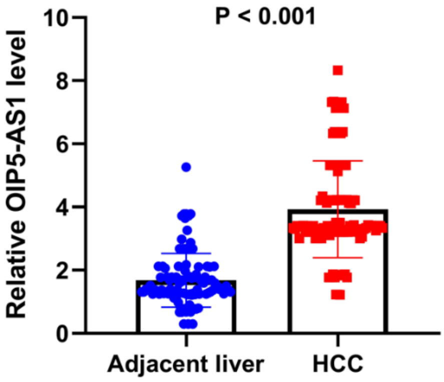

Supplementary figure 6 OIP5-AS1 expression was aberrantly up-regulated in HCC. **a** TCGA database showed that OIP5-AS1 expression was 1.28-fold higher in HCC tissues than that in adjacent non-tumor tissues (P = 0.0026). **b** qRT-PCR analysis of OIP5-AS1 in 102 HCC samples revealed that OIP5-AS1 expression was significantly increased in HCC tissues than adjacent liver tissues.

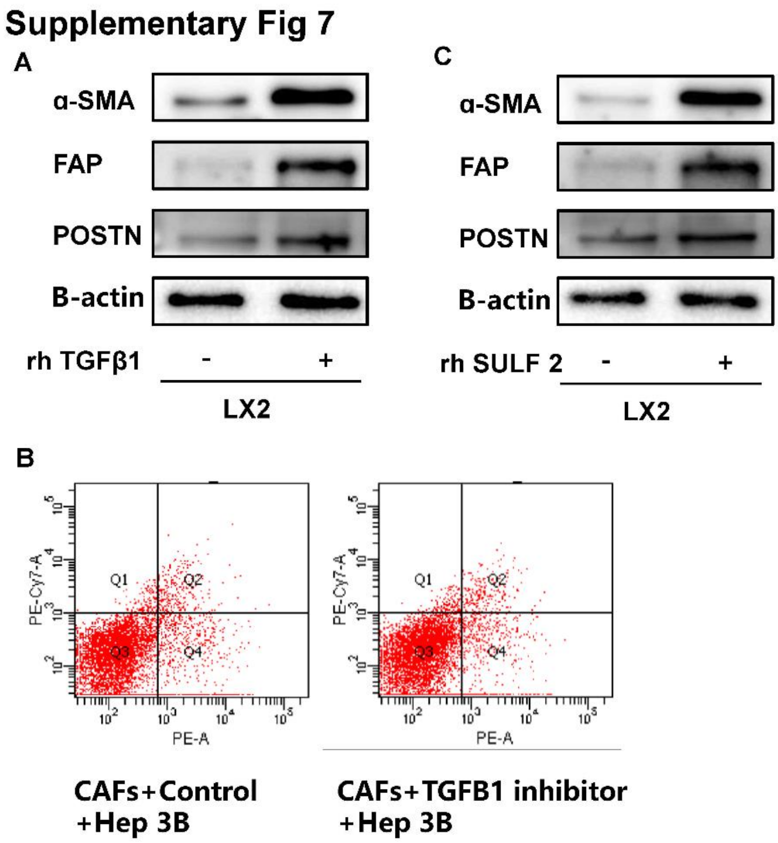

Supplementary figure 7 A Expression levels of α-SMA, FAP and POSTEN of LX2 cells after using rh TGFβ1 were determined by western blotting. B Apoptosis rate of Hep 3B cells co-cultured with CAFs with or without TGFB1 inhibitor. C Expression levels of α-SMA, FAP and POSTEN of LX2 cells after using rh SULF2 were determined by western blotting.

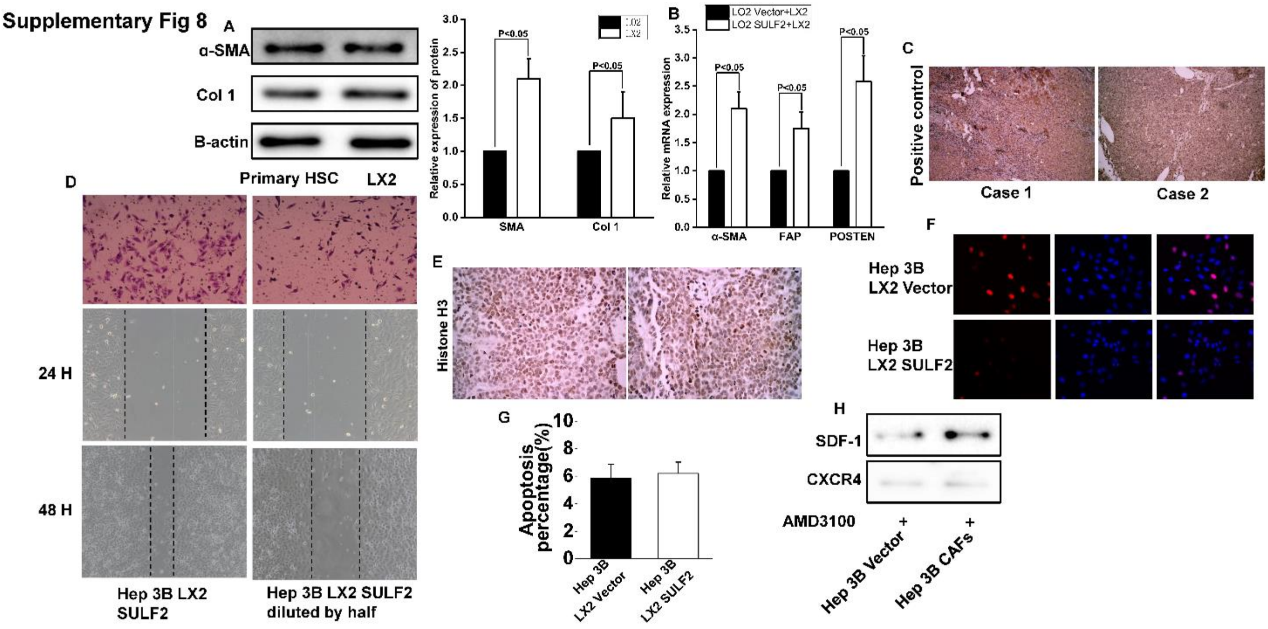

Supplementary figure 8 A Expression levels of α-SMA and Col 1 in Primary HSCs and LX2 cells were determined by western blotting. B qRT-PCR was performed to detect the expression of α-SMA, FAP and POSTEN at mRNA level after LX2 cells co-cultured with LO2 vector or LO2 SULF2. C Positive control for IHC in Fig 1C. D Transwell and Wound healing assay of diluted conditioned medium (dilution factor: dilute in half). E Positive control for IHC in Fig 8D. F Proliferation status of HCC in co-culture systems. G Apoptosis status of HCC in co-culture systems. H The effect of ADM3100 on SDF1 and CXCR4 expression.
