# Supplementary material for: Sulfatase 2-Induced Cancer-Associated Fibroblasts Promote Hepatocellular Carcinoma Progression via Inhibition of Apoptosis and Induction of Epithelial-to-Mesenchymal Transition
Source: Front Cell Dev Biol. 2021 Apr 6;9:631931. doi: 10.3389/fcell.2021.631931 (PMC8056031; doi:10.3389/fcell.2021.631931)

**Supplementary Table 1.** Correlation between the clinicopathologic characteristics and SULF2 expression in hepatocellular carcinoma

| **Clinical features** | | **No. of patients** | **IHC staining for SULF2** | | ***P*** |
| --- | --- | --- | --- | --- | --- |
|  |  |  | **Negative** | **Positive** |  |
| Age | <50 | 39 | 24 | 15 | 0.453 |
|  | ≥50 | 63 | 34 | 29 |  |
| Gender | Male | 81 | 45 | 36 | 0.896 |
|  | Female | 21 | 12 | 9 |  |
| HBV infection | Absent | 29 | 18 | 11 | 0.503 |
|  | Present | 73 | 40 | 33 |  |
| Cirrhosis | Absent | 42 | 28 | 14 | 0.094 |
|  | Present | 60 | 30 | 30 |  |
| AFP level (ng/mL) | <20 | 34 | 11 | 23 | 0.067 |
|  | ≥20 | 68 | 35 | 33 |  |
| Tumor size (cm) | <5 | 34 | 10 | 24 | *0.048 |
|  | ≥5 | 68 | 34 | 34 |  |
| Venous infiltration | Absent | 56 | 17 | 39 | *0.025 |
|  | Present | 46 | 24 | 22 |  |
| TNM stage | I+II | 81 | 29 | 52 | *0.011 |
|  | III+IV | 21 | 14 | 7 |  |

HBV, hepatitis B virus; TNM, tumor-node-metastasis. * indicates P<0.05.

**Supplemental Materials and Methods**

**Supplementary Table 2 List of antibodies,** **reagents and kits used in the study**

| **REAGENTS AND KITS** | | |
| --- | --- | --- |
| **Name** | **Catalog** & **Producer** | **Place** |
| Dulbecco's Modified Eagle's Medium | 8120487，Gibco | Gibco BRL, USA |
| Fetal bovine serum | 2173669,Gbico | Gibco BRL, USA |
| Penicilin Streptomycin | 15140-122,Gbico | Gibco BRL, USA |
| 0.25%Trypsin-EDTA | 25200-056,Gbico | Gibco BRL, USA |
| Cell Culture Inserts | 353097, BD Falcon | BD Biosciences, CA, USA |
| 8-wells Culture slides | 354108, BD Falcon | BD Biosciences, CA, USA |
| co-culture inserts | 363043, BD Falcon | BD Biosciences, CA, USA |
| Annexin V FITC Apoptosis Kit | 556547, BD Falcon | BD Biosciences, CA, USA |
| In Situ Cell Death Detection Kit | 11684817910, Roche | Roche, Germany |
| Caspase 3/7 Assay Kit | ab270771, Abcam | Abcam, USA |
| 5-Ethynyl-2′-deoxyuridine (EdU) assay | C10310, Ribobio | Ribobio, China |
| Matrigel | 354234, Corning® Matrigel® | Corning |
| High Capacity cDNA Reverse Transcription kit | 4374966, Applied Biosystems, | Foster City, CA, USA |
| Protease inhibitor cocktail set III | 539134, Calbiochem | San Diego, CA, USA |
| SYBR® Premix Ex Taq™ II (Perfect Real-Time) kit | 74104, Qiagen | Takara Bio, Otsu, Japan |
| Phenylmethylsulfonyl fluoride (PMSF) | FNN0011, Invitrogen | Grand Island, NY, USA |
| ECL-enhanced chemiluminescence reagents | Denville Scientific Inc. | Metuchen, NJ, USA |
| 4-15% Tris HCl gel | BioRad | Richmond, CA, USA |
| Cell Extraction Buffer | FNN0011, Life technologies | Staley Road Grand Island, NY |
| DAKO kit for IHC | K4010, DAKO, Inc. | Carpinteria, CA, USA |
| Recombinant SULF2 |  |  |
| TGFB1 inhibitor | LY-364947, Peprotech | Peprotech, China |
| Recombinant TGFβ1 | 240-B-010, R&D | Minneapolis, MN, USA |
| Formaldehyde Solution | F1635, Sigma Aldrich | Sheboygan Falls, WI |
| Dynabeads Protein G | 10004D, Thermo Fisher Scientific | Waltham, MA |
| PerfeCTa SYBR Green FastMix | Quanta BioSciences Inc. | Gaithersburg, MD |

| **ANTIBODIES** | | | | |
| --- | --- | --- | --- | --- |
| **Antibodies** | **suppliers** | **concentration** | **temperature and timing of incubation** | **analyses (WB or IHC)** |
| SULF2 | #PA5-43331, ThermoFisher SCIENTIFIC | 1:1000 | 4 ℃, overnight | WB |
| ɑ-SMA | #19245, Cell Signaling Technology(CST, Danvers, MA, USA) | 1:1000 for WB  1:400 for IHC | 4 ℃, overnight | WB, IHC |
| FAP | #66562, Cell Signaling Technology(CST, Danvers, MA, USA) | 1:1000 for WB  1:100 for IHC | 4 ℃, overnight | WB, IHC |
| POSTN | #91771, Cell Signaling Technology(CST, Danvers, MA, USA) | 1:1000 for WB  1:100 for IHC | 4 ℃, overnight | WB, IHC |
| TGFβ1 | #3711, Cell Signaling Technology(CST, Danvers, MA, USA) | 1:1000 | 4 ℃, overnight | WB |
| p-Smad3 | #9520, Cell Signaling Technology(CST, Danvers, MA, USA) | 1:1000 | 4 ℃, overnight | WB |
| SDF-1 | #3530, Cell Signaling Technology(CST, Danvers, MA, USA) | 1:1000 | 4 ℃, overnight | WB |
| CXCR4 | #997680, Cell Signaling Technology(CST, Danvers, MA, USA) | 1:1000 | 4 ℃, overnight | WB |
| p-PI3K | #4228, Cell Signaling Technology(CST, Danvers, MA, USA) | 1:1000 | 4 ℃, overnight | WB |
| p-Akt | #4060, Cell Signaling Technology(CST, Danvers, MA, USA) | 1:1000 | 4 ℃, overnight | WB |
| p-BAD | #5284, Cell Signaling Technology(CST, Danvers, MA, USA) | 1:1000 | 4 ℃, overnight | WB |
| p-Caspase9 | P55211, Bioworld | 1:1000 | 4 ℃, overnight | WB |
| Collgea-1α | #72026, Cell Signaling Technology(CST, Danvers, MA, USA) | 1:1000 | 4 ℃, overnight | WB |

**Supplemental Materials and Methods**

**Supplementary Table 3** **The sequences of the primers for qRT-PCR.**

| Gene name | Primer sequence |
| --- | --- |
| Human SULF2 F | 5’- CGGAACGGGGTGAAAGAGAA -3’ |
| Human SULF2 R | 5’- TGATCCAGTGTTTGTCCGGG -3’ |
| Human OIP5-AS1 F | 5′- CCTGGCTCATCCTGTGACATA -3′ |
| Human OIP5-AS1 R | 5′- TGGCACTGCATGAGGGATTTT -3′ |
| Human miR153-3p F | 5′- CGGCTGCCAGCGTCATTTTT -3′ |
| Human miR153-3p R | 5′-GGCCACACCTGCCAGTGAT-3′ |
| Human α-SMA F | 5′-AAATGAACGTTTCCGCTGCC-3′ |
| Human α-SMA R | 5′-CTTCATGGTGCTGGGTGCTA-3′ |
| Human FAP F | 5’- TCCTGGCTTCAGCTTCCAAC -3’ |
| Human FAP R | 5’- TTTACTCCCAACAGGCGACC -3’ |
| Human POSTN F | 5’- AAGCGCTTTAGCACCTTCCT -3’ |
| Human POSTN R | 5’- CTTCCTCACGGGTGTGTCTC -3’ |
| Human TGFβ1 F | 5’- CGACTCGCCAGAGTGGTTAT -3’ |
| Human TGFβ1 R | 5’- CGGTAGTGAACCCGTTGATGT -3’ |
| Human SDF-1 F | 5’- TGAGCTACAGATGCCCATGC -3’ |
| Human SDF-1 R | 5’- CCACTTTAGCTTCGGGTCAA -3’ |
| Human β-actin F | 5′-GTGAAGGTGACAGCAGTCGGTT-3′ |
| Human β-actin R | 5′- GAAGTGGGGTGGCTTTTAGGA -3′ |

**Supplemental Subjects and Methods**

**1.1 5-Ethynyl-2′-deoxyuridine (EdU) assay**

Proliferating cells were assessed using a 5-ethynyl-2-deoxyuridine (EdU) Labeling/Detection Kit (RiboBio), in accordance with the manufacturer's protocol. Briefly, Hep 3B co-cultured with LX2 Vector or LX2 SULF2 were cultured in 96-well plates at 5 × 10^3^ cells per well. Then, 50 μmol/L EdU labeling media were added to the 96-well plates, and they were incubated for 2 hours at 37°C under 5% CO2. After treatment with 4% paraformaldehyde and 0.5% Triton X-100, cells were stained with anti-EdU working solution. DAPI was used to label cell nuclei. The percentage of EdU-positive cells was calculated from five random fields in three wells after analyses of fluorescent microscopy.

**1.2 Isolation of primary HSCs**

The methods used to isolate HSCs from HCC tissue have been previously described [1]. Briefly, surgically resected liver peritumoral tissues were minced into 2–3-mm fragments, washed in phosphate-buffered saline (PBS), and cultured in 6-well plates supplemented with F12/DMEM containing 10% fetal bovine serum and 1% penicillin/streptomycin. primary HSCs were allowed to grow out of the tissue fragments at 37 °C with 5% CO2. After purification, the expression of α-SMA and Col 1 in these cells was determined by western blotting. We used HSCs within 6 passages.

**References**

1. Santamato A, Fransvea E, Dituri F, Caligiuri A, Quaranta M, Niimi T, et al. Hepatic stellate cells stimulate HCC cell migration via laminin-5 production. Clinical Science. 2011 Apr 28;121(4):159–68.

**Certificate for editing**


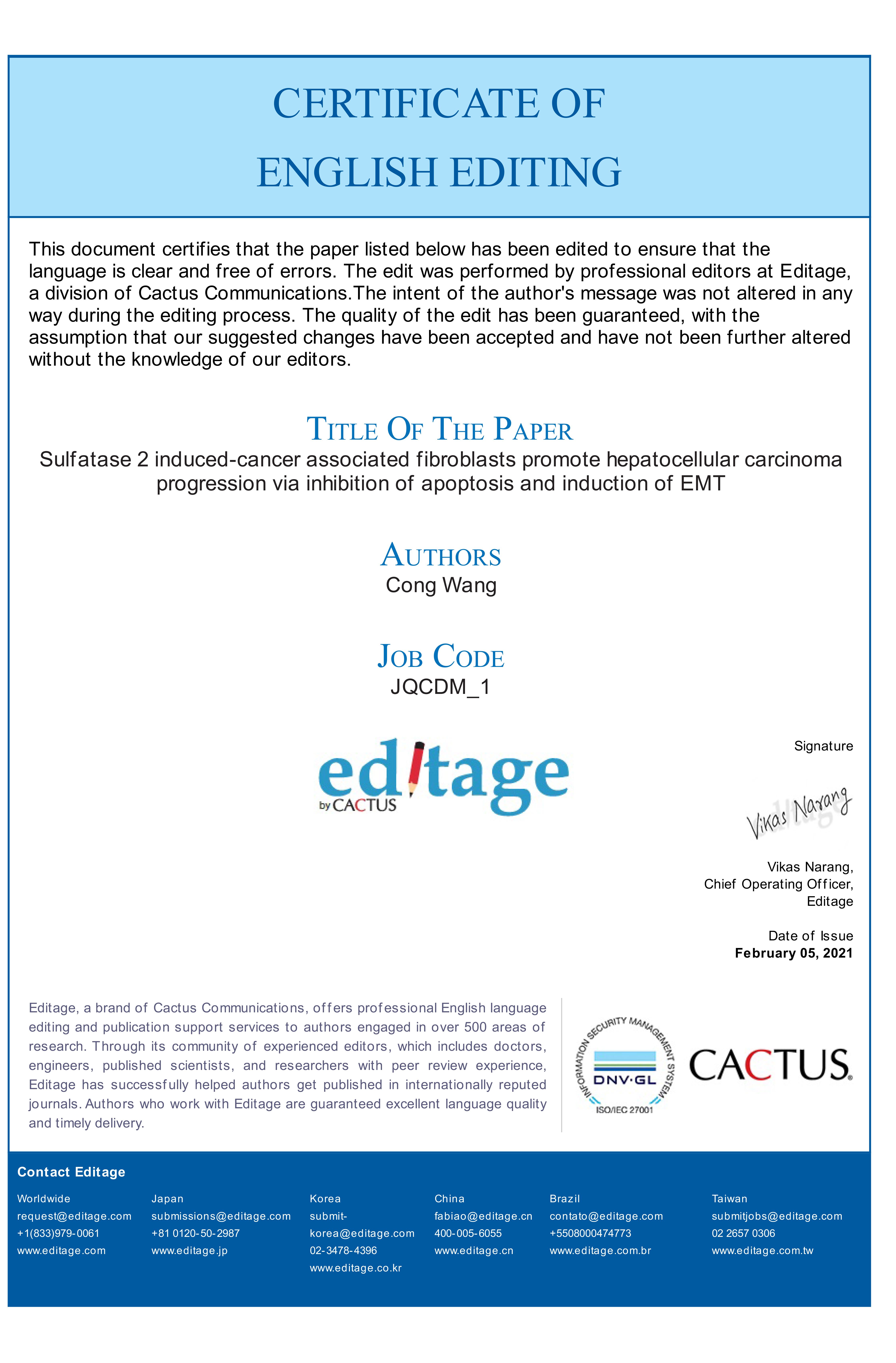

Supplement: Supplementary file 2 [file Table_1.docx]
